# Functional redundancy of HSPA1, HSPA2 and other HSPA proteins in non-small cell lung carcinoma (NSCLC); an implication for NSCLC treatment

**DOI:** 10.1038/s41598-019-50840-7

**Published:** 2019-10-07

**Authors:** Damian Robert Sojka, Agnieszka Gogler-Pigłowska, Natalia Vydra, Alexander Jorge Cortez, Piotr Teodor Filipczak, Zdzisław Krawczyk, Dorota Scieglinska

**Affiliations:** 1 Center for Translational Research and Molecular Biology of Cancer, Maria Sklodowska-Curie Institute – Oncology Center Gliwice Branch, 44-101 Gliwice, Poland; 2 Lung Cancer Program, Lovelace Respiratory Research Institute, Albuquerque, New Mexico 87108 USA

**Keywords:** Non-small-cell lung cancer, Cancer therapeutic resistance

## Abstract

Heat shock proteins (HSPs) are a large group of chaperones considered critical for maintaining cellular proteostasis. Their aberrant expression in tumors can modulate the course of processes defined as hallmarks of cancer. Previously, we showed that both stress-inducible HSPA1 and testis-enriched HSPA2, highly homologous members of the HSPA (HSP70) family, are often overexpressed in non-small cell lung carcinoma (NSCLC). HSPA1 is among the best characterized cancer-related chaperones, while the significance of HSPA2 for cancer remains poorly understood. Previously we found that in primary NSCLC, HSPA1 was associated with good prognosis while HSPA2 correlated with bad prognosis, suggesting possible different roles of these proteins in cancer. Therefore, in this work we investigated the impact of HSPA1 and HSPA2 on NSCLC cell phenotype. We found that neither paralog-selective nor simultaneous knockdown of HSPA1 and HSPA2 gene expression reduced growth and chemoresistance of NSCLC cells. Only blocking of HSPA proteins using pan-HSPA inhibitors, VER-155008 or JG-98, exerted potent anticancer effect on NSCLC cells, albeit the final outcome was cell type-dependent. Pan-HSPA inhibition sensitized NSCLC cells to bortezomib, but not to platinum derivates. Our result suggests the inhibitors of proteasome and HSPAs seem an effective drug combination for pre-clinical development in highly aggressive NSCLC.

## Introduction

Heat shock proteins (HSPs) are a large group of chaperones, which are considered critical for maintaining cellular protein homeostasis. On the basis of amino acids sequence similarity and molecular weight, HSPs are sub-grouped into several families^1^. HSPs localize in various subcellular compartments, and they are involved in multiple processes, including apoptosis, senescence, autophagy, immunoresponse and many others. Some of HSPs can perform cell-type specific roles^2^. Dysregulated expression of HSPs in cancer cells can manifest by increased production of the proteins and/or their abnormal intracellular localization. The results of multiple studies justify conclusion that altered expression of HSPs can influence all processes defined as hallmarks of cancer. In particular, HSPs having anti-apoptotic and proteostasis-controlling function, can modulate the development and progression of cancer and impair the effectiveness of chemotherapy^3,4^. Recently, HSPs are being considered as a “druggable target” for the development of a novel anticancer drugs^5,6^.

The observation that the pattern of HSPs expression can alter during tumor development initiated a number of studies aimed at clarifying a potential predictive and prognostic value of HSPs^7,8^. In general, only a few HSPs have been examined in terms of their value as a potential tumor marker. Recent comprehensive summaries revealed that although there is a strong conviction that HSPs may be useful as tumor markers and/or therapeutic targets, at the current stage of research an unambiguous assessment of their true clinical value is still missing^4,9^. A lack of consensus may result either from the strong influence of molecular background or tumor microenvironment on HSPs functionality^10^, from focusing on the individual contribution of a single HSP, instead of several similar HSPs isoforms, or from omitting from the investigation those HSPs, whose significance remains unperceived.

One of poorly characterized HSPs is a testis-enriched HSPA2 protein, a member of the HSPA (HSP70) multigene family considered critical for men’s fertility^11,12^. The human *HSPA2* gene, beside spermatogenic cells, is also expressed in some somatic tissues in a cell-type-specific manner. Specifically, the high level of HSPA2 was confined to various stratified and pseudostratified epithelia^13^. Although HSPA2 is overexpressed in various tumors^14^, a potential prognostic value of HSPA2 has been studied in only few tumor types. The available evidence indicates that HSPA2 may have different prognostic value than HSPA1, a major stress-inducible and the most thoroughly investigated chaperone from the HSPA (HSP70) family, also frequently over-represented in cancer. In esophageal and pancreatic cancers a high expression of HSPA2 correlates with poor survival in patients^15–17^, while the opposite association was reported for HSPA1^18–20^. In breast tumors conversely, a positive prognostic value was found for HSPA2^21^, but negative for HSPA1^22,23^. In our earlier studies we found that prognostic values of HSPA2 and HSPA1 expression in patients with primary non-small cell lung carcinoma (NSCLC) are opposite. Immunohistochemical analysis performed on the same set of postsurgical samples revealed that a high expression of HSPA2 correlates with poor prognosis, while HSPA1 correlates with good outcomes^14,24^. Importantly, our findings correspond well to results showing negative prognostic value of a decreased expression of HSPA1 in small cell lung carcinoma^25^, or association between a high level of HSPA1 and longer disease-free survival of NSCLC patients who received adjuvant platinum-based chemotherapy^26^.

Lung cancer, with the most common NSCLC subtype, remains the leading cause of cancer-related death. The most common treatment options for NSCLC are surgery, radiotherapy and platinum-based doublet chemotherapy. A search for novel therapy regimens that would improve efficacy of anticancer treatments pointed out potential beneficial effects of proteasome inhibitors. The first proteasome inhibitor tested in clinical trials for NSCLC treatment was bortezomib (BTZ). Recent summary of clinical results shows rather modest anticancer activity of BTZ in therapy of solid tumors^27^. Nevertheless, *in vitro* studies showed that BTZ can potentiate the anticancer effect of cisplatin (CDDP) on various NSCLC cell lines, what encourages further investigations^27–29^.

So far, studies aimed at understanding the impact of HSPs on the effectives of lung cancer treatment have concentrated on the HSP90 (HSPC) protein, mainly due to development of multiple inhibitors. Findings from clinical trials aimed at testing HSPC inhibitors for NSCLC therapy reported promising results^30,31^. Importantly, in NSCLC cells, HSPC inhibitors enhanced antitumor activity of CDDP^32,33^, and BTZ^34^. With regard to the HSPA proteins, the knowledge of their impact on tumor cell proliferation and sensitivity to CDDP and BTZ is rather minor. *In vitro* studies performed on NSCLC cell lines such as A549 and H460 showed that both RNAi-mediated silencing of HSPA1 expression or chemical inhibition of HSPA function led to reduced cell proliferation^35,36^. However, in another study siRNA-mediated depletion of HSPA1 in A549 cells had no effect on viability, albeit sensitized cells to CDDP^37^. As for HSPA2, its potential impact on growth and resistance to CDDP, BTZ and other anticancer drugs has not been tested in NSCLC cells.

Bearing in mind that HSPA1 and HSPA2 can be expressed in NSCLC cells either together or separately and may have a different prognostic value, we found important to study the influence of both proteins on proliferation rate and chemoresistance of NSCLC cells to CDDP and BTZ. In this work we found that HSPA proteins, as a group of redundant factors support proliferation and contribute to resistance of NSCLC cells to proteasome inhibitors.

## Results

### The endogenous level of HSPA isoforms and HSPC shows no correlation with sensitivity of NSCLC cells to CDDP

**Figure 1:**
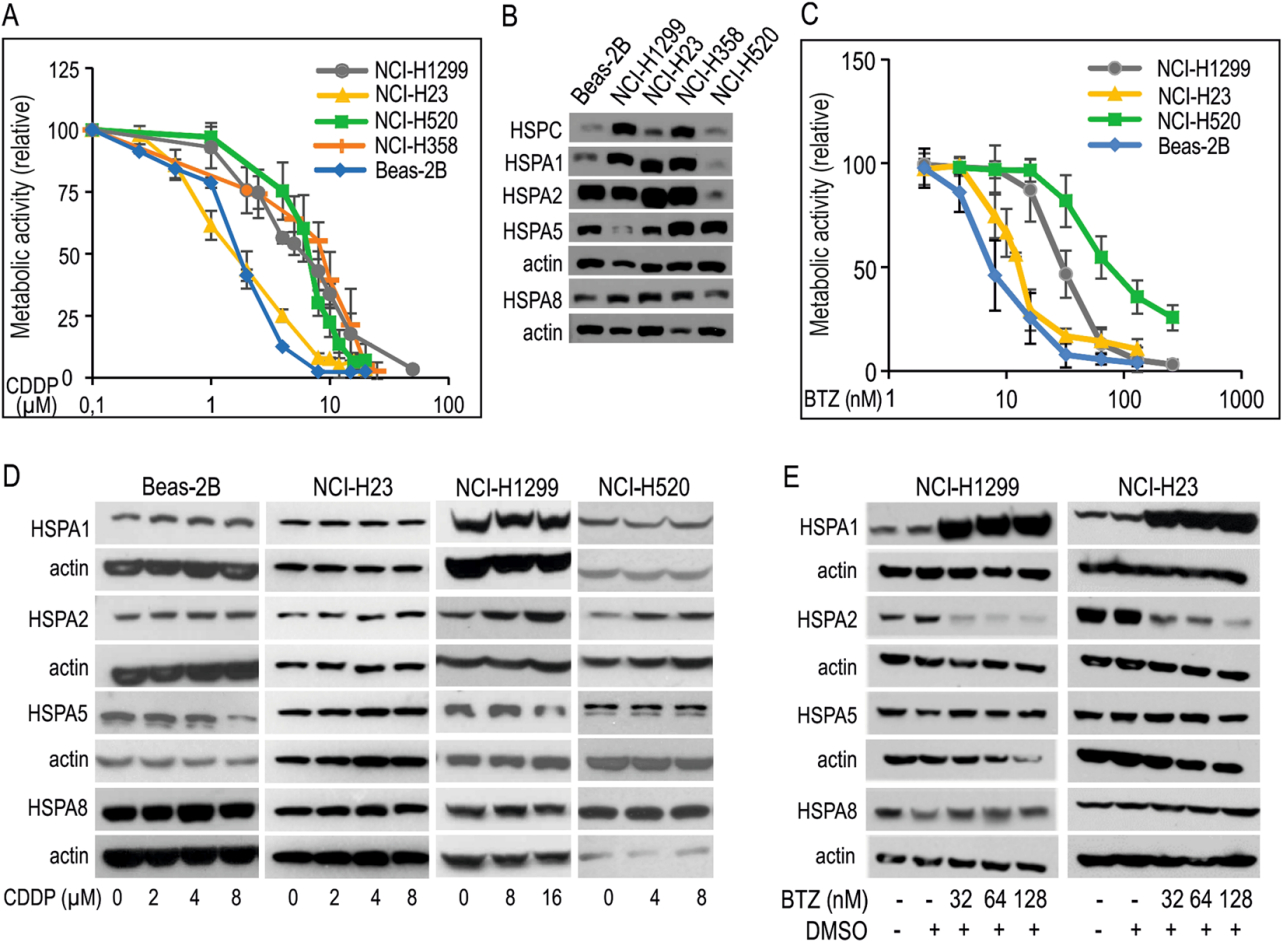
Dose response curves of cisplatin (CDDP) and bortezomib (BTZ) in NSCLC cell lines and immortal bronchial epithelial cells (Beas-2B) differing in endogenous and drug-induced levels of HSPA proteins expression. (**A**) Graphical representation of cell viability following 72 h treatment with CDDP measured by MTS assay (for each dose three independent repeats were performed, each in triplicate). (**B**) The basal expression of HSPA1, HSPA2, HSPA5, HSPA8 and HSPC protein. Representative immunoblots are shown (n = 3), actin is used as a protein loading control. (**C**) Graphical representation of cell viability following 72 h treatment with various concentrations of BTZ measured by MTS assay (for each dose at least three independent repeats were performed, each in triplicate). (**D**,**E**) Expression of HSPA proteins in cells exposed to CDDP (**D**) or BTZ (**E**) for 24 h. Representative immunoblots are shown (n = 4), actin was used as a protein loading control.

One of essential question relevant to lung cancer chemotherapy that has not been unequivocally answered yet, is to what extent HSPA proteins contribute to anticancer drug resistance. Here, we juxtaposed sensitivity of human NSCLC cell lines and one immortal bronchial epithelial Beas-2B cell line to CDDP and BTZ with the endogenous expression of cancer-related HSPA proteins (Fig. 1). IC_50_ values of CDDP and BTZ calculated for each cell line are shown in Table 1. We analyzed the expression of the HSPA family member, namely HSPA1, HSPA2 and HSPA8, cytosolic/nuclear chaperones and HSPA5, the endoplasmic reticulum-located, as well as HSPC (HSP90) protein in CDDP-resistant (NCI-H1299, NCI-H358, NCI-H520) and CDDP-sensitive (Beas-2B, NCI-H23) cell lines (Fig. 1A,B). We found that there is no clear relationship between the endogenous levels of HSPAs and cell susceptibility to CDDP (Fig. 1A,B). Though NCI-H520 cells contained lower levels of HSPA1, HSPA2 and HSPA8 than NCI-H1299 and NCI-H358 cells (Fig. 1B), all these cell lines grouped as CDDP-resistant ones (Fig. 1A, Table 1). Also, CDDP-resistant (NCI-H1299, NCI-H358) and CDDP-sensitive (NCI-H23, Beas-2B) cells contained similar levels of HSPA1, HSPA2 and HSPA8 and similarly, no correlation between HSPA5 expression and cell sensitivity to CDDP was evident (Fig. 1). Also, no clear relationship between the basal levels of HSPAs, HSPC and cell sensitivity to BTZ could be observed. As expected, viability of cells exposed to BTZ decreased in a dose-dependent manner (Fig. 1C). Beas-2B and NCI-H520 cell lines were the most and the least sensitive ones, respectively (Fig. 1C). Although the IC_50_ value of BTZ for NCI-H23 cells was lower than for NCI-H1299 cells (Table 1), both cell lines contained comparable levels of HSPA1, HSPA2 and HSPA8 proteins (Fig. 1B).

**Table 1:** IC_50_ value calculated for cisplatin, bortezomib, VER-155008 and JG-98.

Cell line	Cisplatin IC50 µM (95% confidence interval)	Bortezomib IC50 nM (95% confidence interval)	VER-155008 IC50 µM (95% confidence interval)	JG-98 IC50 µM (95% confidence interval)
Beas-2B	1.62 (1.45–1.79)	6.48 (5.78–7.26)	>40	no data
NCI-H23	1.56 (1.43–1.69)	11.12 (10.43–11.88)	5.11 (4.6–5.74)	0.32 (0.3–0.34)
NCI-H1299	5.22 (4.75–5.73)	30.44 (28.55–32.51)	5.91 (5.52–6.31)	1.63 (1.43–1.89)
NCI-H358	6.83 (6.09–7.56)	no data	no data	no data
NCI-H520	5.96 (5.5–6.44)	72.1 (66.59–78.11)	13.84 (12.84–14.96)	no data

Given that expression of HSPAs can be induced by various cellular stressors including anticancer drugs, we tested whether CDDP and BTZ can stimulate the expression of HSPA isoforms. We observed that 24 h treatment with the increasing doses of CDDP had no effect on the expression of HSPA1 and HSPA8 proteins; slight inhibitory effect on HSPA5 expression in NCI-H1299 and Beas-2B cells, and stimulatory effect on HSPA2 expression in the CDDP-resistant cells (Fig. 1D). These observation showed that CDDP can differentially modulate expression of HSPA2 (increase) and HSPA5 (decrease) in a cellular context-dependent manner.

In contrast, treatment of NSCLC cells for 24 h with increasing doses of BTZ (32–128 nM) highly induced the level of HSPA1 and substantially decreased the level of HSPA2, without affecting the expression of HSPA5 and HSPA8 (Fig. 1E). Altogether, our results allow to speculate that NSCLC cells might require increased levels of HSPA2, but not other HSPAs, to cope with CDDP-induced stress, as well as the cells might need higher levels of HSPA1, but not other HSPAs, to counteract BTZ-induced proteotoxic stress.

### Paralog-specific knockdown of HSPA1 and HSPA2 in NSCLC cells has no effect on proliferation, clonogenic potential and sensitivity to platinum derivatives and BTZ

**Figure 2:**
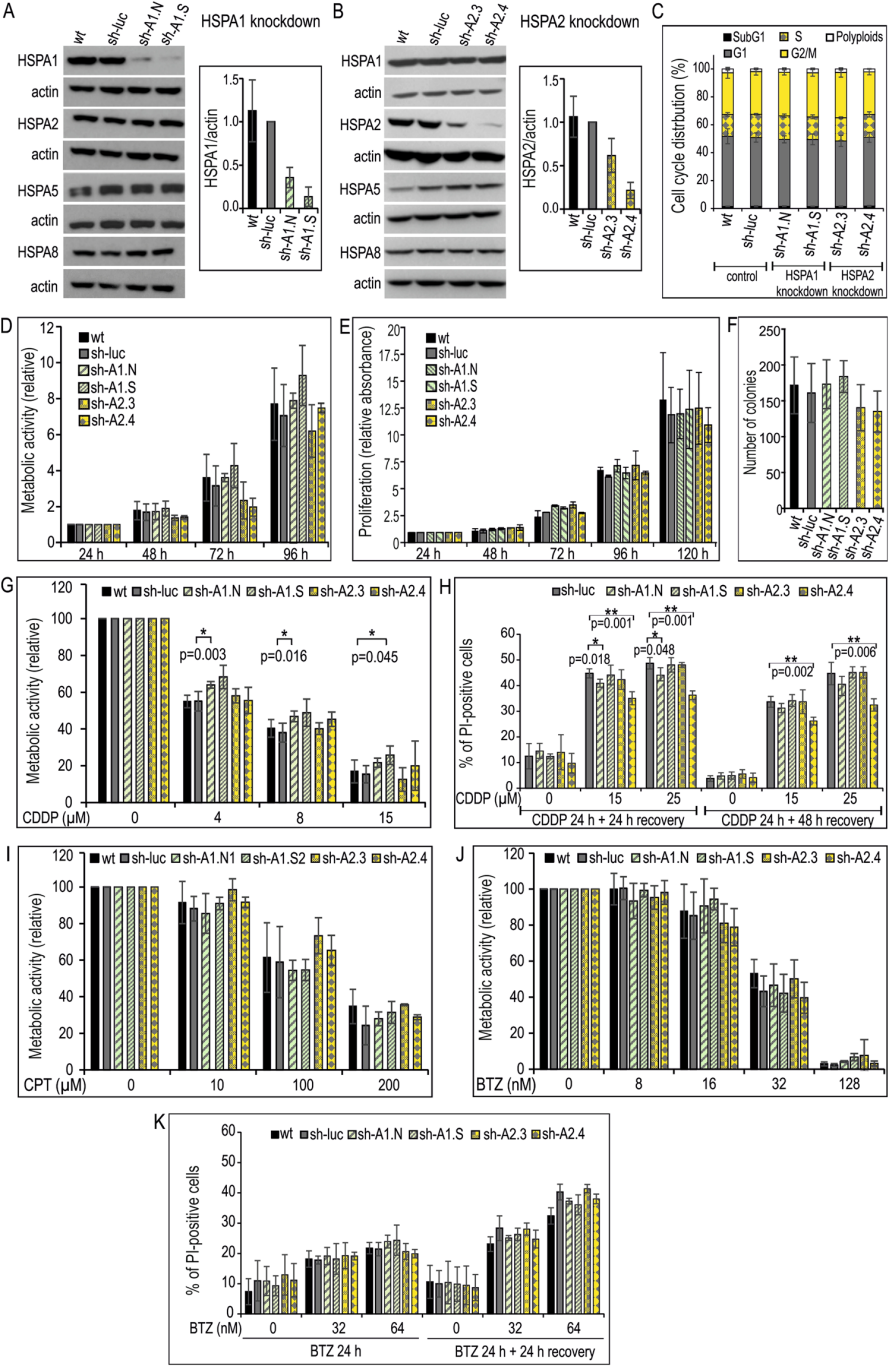
Effects of HSPA1 or HSPA2 depletion on the proliferation, clone-forming ability and chemoresistance of NCI-H1299 cells. (**A**,**B**) Levels of HSPA proteins in wild-type (wt); control sh-luc cells stably transduced with a non-targeting shRNA-luc sequence; sh-A1.N and sh-A1.S cell lines stably transduced with HSPA1-targeting sh-RNA-A1.N or sh-RNA-A1.S sequences, respectively (**A**); sh-A2.3 and sh-A2.4 cell lines stably transduced with HSPA2-targeting sh-RNA-A2.3 or sh-RNA-A2.4 sequences, respectively (**B**). Representative immunoblots are shown (n = 3), actin was used as a protein loading control. Graph shows results of densitometric analysis of HSPA1 (**A**) or HSPA2 (**B**) immunodetection (HSPA1 n = 3, HSPA2 n = 7). (**C**) Cell cycle phase distribution in sub-confluent cells at 48 hours (h) after plating. Graph shows percentage of cells (mean ± SD, n = 3, each in two technical replicas). (**D**) Cell proliferation assessed by MTS assay. Results are expressed as mean ± SD (n = 3, each in three technical replicas) in relation to values obtained at 24 h after plating. (**E**) Results of proliferation assay (n = 3, each in six technical replicas) assessed using crystal violet staining. Relative absorbance of stained cells was plotted against time (24–120 h) of continuous growth. (**F**) Number of colonies formed by cells plated onto 6-well dishes (1 × 10^3^ cells/well) and cultured for 7–8 days. Colonies were counted manually (mean ± SD, n = 5 each in three technical replicas). (**G**,**K**) Effects of HSPA1 or HSPA2 depletion on resistance of cells to cisplatin (CDDP) (**G**,**H**), carboplatin (CPT) (I) or bortezomib (BTZ) (J-K). Cell viability was measured using MTS assay after 72 h treatment (**G,I,J**). Results are expressed relative to untreated control (mean ± SD from at least three independent experiments, each in triplicate, *p < 0.05, statistical significance was determined by two-tailed t-test). Cell death detection using propidium iodide (PI) uptake test after 24 h treatment with CDDP (**H**) or BTZ (**K**) and/or following 24 or 48 h growth without drugs. Results show mean values ± SD from two (**H**) or three (**K**) independent repeats, each at least in duplicate. Statistical significance was determined using two-tailed t-test.

Our primary objective was to examine whether deficiency in HSPA1 or HSPA2 would have an effect on proliferation of NSCLC cells. For this purpose stable knockdown of either HSPA1 or HSPA2 by specific shRNA (Table 2) was established in NCI-H1299 and NCI-H23 cell lines. As a control we used cells stably transduced with a lentiviral vector that contained non-targeting shRNA (sh-luc cell line). Importantly, the level of HSPA2 and HSPA1 proteins in sh-luc cells was similar to that observed in wild-type (wt) cells, both in NCI-H1299 (Fig. 2A,B) and NCI-H23 (Fig. S1A,B) cell lines. Effective reduction in HSPA1 or HSPA2 levels was achieved with the use of sh-A1.N and sh-A1.S sequences (Figs 2A, S1A) or sh-A2.3 and sh-A2.4 sequences (Figs 2B, S1B), respectively. The silencing was specific for the target gene, as the expression levels of other highly homologous HSPA proteins remained unaffected in HSPA1 (Figs 2A, S1A) and HSPA2-deficient (Figs 2B, S1B) cells.

**Table 2:** Sequence of shRNA particles.

Gene	Sequence	Method
HSPA1	sh-A1.N 5′-CACCGTGTTTGACGCGAAG-3'	RNAi-mediated silencing
HSPA1	sh-A1.S 5′-GAAGGACGAGTTTGAGCACAA-3'	RNAi-mediated silencing
HSPA2	sh-A2.3 5′-GCCATCCTCATCGGCGACAAA-3′	RNAi-mediated silencing
HSPA2	sh-A2.4 5′-GCGGCGAGAAGAACGTGCTCAT-3′	RNAi-mediated silencing
LUC	sh-luc 5′-GTGCGTTGCTAGTACCAAC-3′	RNAi-mediated silencing

Analysis of sub-confluent cells cultured for 48 h after plating did not reveal significant changes in the fraction of cells in the different cell cycle phases between control (sh-luc, wt), HSPA1-deficient (sh-A1.N, sh-A1.S) and HSPA2-deficient (sh-A2.3, sh-A2.4) cells (Figs 2C, S1C). Using MTS assay and the crystal violet staining method we found that the control, HSPA1- and HSPA2-deficient cells showed similar metabolic activity and proliferation rates, respectively for up to 96–120 h of continuous culture (Figs 2D,E, S1D,E). Also, the control, HSPA1- and HSPA2-deficient cells formed a comparable number of colonies (Figs 2F, S1F). These results showed, that neither specific knockdown of HSPA1 nor HSPA2 had noticeable impact on proliferation and clone forming ability of NCI-H1299 and NCI-H23 cells.

Our second objective was to examine whether isoform-specific depletion of HSPA1 or HSPA2 would have an effect on resistance of NSCLC cells to platinum derivatives, CDDP and carboplatin (CPT), or proteasome inhibitor, BTZ. As expected, direct exposure of NCI-H1299 and NCI-H23 cells, both control and deficient in HSPA1 or HSPA2, to CDDP or CPT caused a dose-dependent reduction of metabolic activity (Figs 2G,I, S1G,I). However, 72 h treatment with CDDP evoked similar reduction of metabolic activity in the control sh-luc, sh-A1.S (HSPA1-deficient), sh-A2.3 and sh-A2.4 (HSPA2-deficient) cells (Figs 2G, S1G). Analysis of direct cytotoxic effect of CDDP (24 h, 15 µM, 25 µM) performed using propidium iodide (PI) uptake test showed that deficit in HSPA2 or HSPA1 did not sensitize NSCLC cells to the drug. Comparing with sh-luc cells, the numbers of PI-positive dead cells were similar or lower in cells deficient in HSPA1 or HSPA2 at 24 h or 48 h after 24 h treatment with CDDP (Figs 2H, S1H). In the case of NCI-H1299, HSPA1-deficient sh-A1.N cells and HSPA2-deficient sh-A2.4 cells were even slightly more resistant to CDDP than sh-luc cells (Fig. 2G,H). In turn, for both HSPA1- or HSPA2-deficient sublines, as well as for control cells the range of growth inhibition after CPT treatment, was similar (Figs 2I, S1I). Collectively, our results showed that deficiency in HSPA1 or HSPA2 proteins did not sensitized NSCLC cells to platinum derivatives.

Next we investigated whether specific knockdown of HSPA1 or HSPA2 could sensitize NSCLC cells to BTZ. We found that exposure of NCI-H1299 (BTZ-resistant) and NCI-H23 (BTZ-sensitive) cells, both control and HSPA1- or HSPA2-deficient sublines to BTZ caused a dose-dependent reduction of cell viability (Figs 2J,K, S1J,K), although differences in the extent of growth inhibition and cell death between treated cell lines were not significant (Figs 2J,K, S1J,K).

### Combined knockdown of HSPA1 and HSPA2 isoforms in NCI-H1299 cells has no effect on proliferation and sensitivity to BTZ but may increase the resistance to CDDP

**Figure 3:**
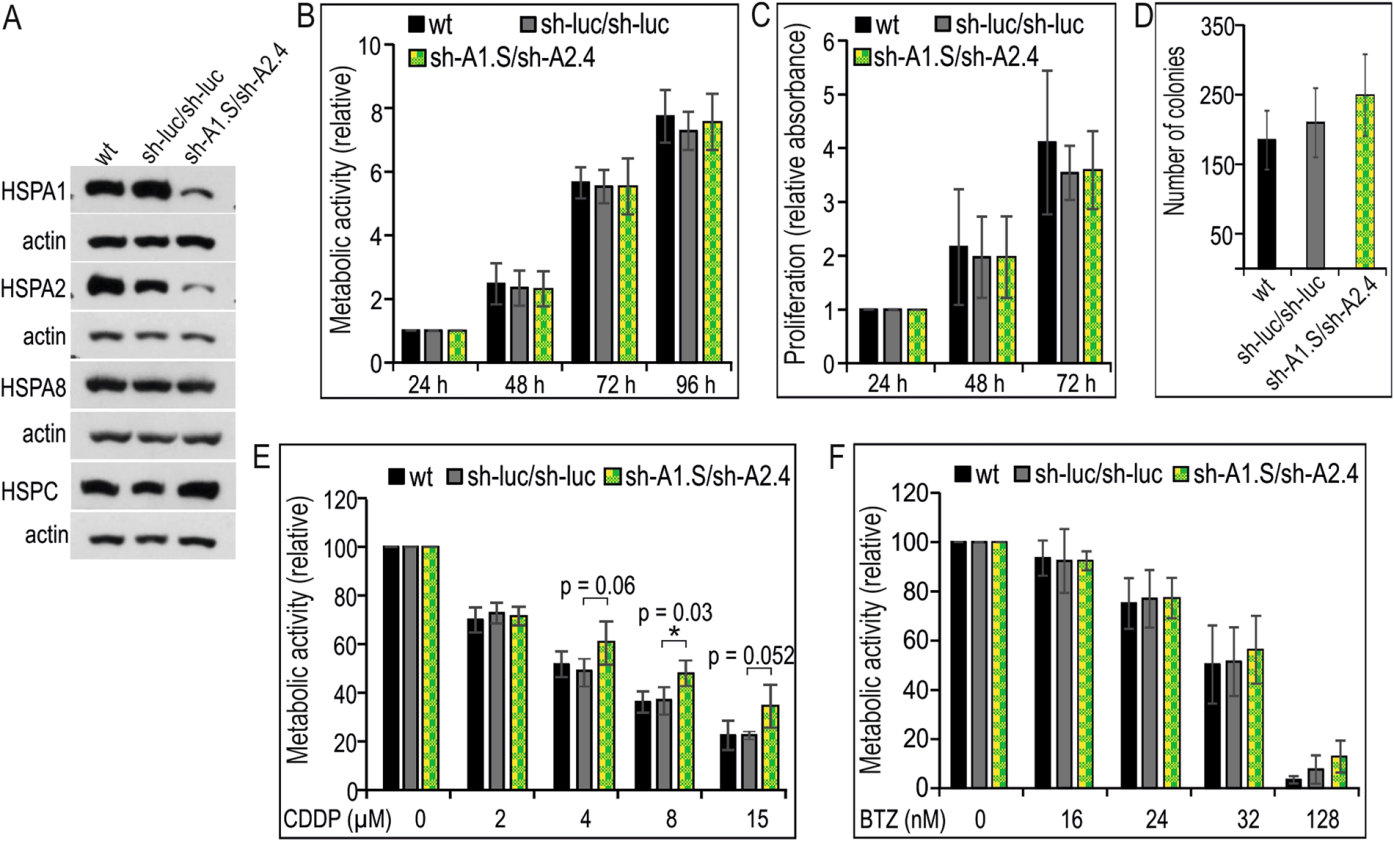
Effects of combined HSPA1 and HSPA2 deficiency on the proliferation, clone-forming ability and chemoresistance of NCI-H1299 cells. (**A**) Levels of HSPAs and HSPC in wild-type (wt); control sh-luc/sh-luc cells doubly transduced with a non-targeting shRNA-luc sequence; sh-A1.S/sh-A2.4 cell line simultaneously transduced with HSPA1- and HSPA2-targeting sequences. Representative immunoblots are shown (n = 3), actin was used as a protein loading control. (**B**) Cell proliferation assessed using MTS assay. Results are expressed as mean ± SD (n = 5, each in three technical replicas) in relation to values obtained at 24 h after plating. (**C**) Results of crystal violet staining proliferation assay (n = 4, each in three technical replicas). Relative absorbance of stained cells was plotted against time (24–72 h) of continuous growth. (**D**) Number of colonies formed by cells plated onto 6-well dishes (1 × 10^3^ cells/well) and cultured for 7–8 days. Colonies were counted manually (mean ± SD, n = 7 each in three technical replicas). (**E**,**F**) Effects of simultaneous HSPA1 and HSPA2 deficiency on resistance of cells to cisplatin (CDDP) (**E**) or bortezomib (BTZ) (**F**). Cell viability was measured using MTS assay after 72 h treatment. Results are expressed relative to untreated control (mean ± SD, n = 4, each in triplicate, *p < 0.05, statistical significance was determined by two-tailed t-test).

The results presented above showed that neither endogenous HSPA1 nor HSPA2 proteins played a significant role in resistance of NSCLC cells to BTZ. This suggested that these two proteins may have a redundant role for NSCLC cells growth and chemoresistance. Therefore, we transduced NCI-H1299 cells with sh-A1.S and sh-A2.4 shRNA sequences in order to establish stable cell lines deficient in both HSPA1 and HSPA2 proteins (sh-A1.S/sh-A2.4 cell line). As a control we used cells transduced twice with a vector encoding non-targeting shRNA (sh-luc/sh-luc cell line). We achieved effective and specific reduction in both HSPA1 and HSPA2 levels (Fig. 3A). The expression of HSPA8 (highly homologous) and HSPC protein remained unaffected in double knockdown cells (Fig. 3A).

Using MTS assay and the crystal violet staining method we found that metabolic activity and proliferation rate during 96 h of continuous culture (Fig. 3B,C), as well clone forming ability (Fig. 3D) of sh-luc/sh-luc and sh-A1.S/sh-A2.4 cells were comparable. Thus, our results showed that simultaneous knockdown of HSPA1 and HSPA2 isoforms had no effect on NCI-H1299 cell growth.

Next, we studied if double knockdown of HSPA1 and HSPA2 would affect the resistance of NCI-H1299 cells to CDDP or BTZ. Using MTS assay we found that treatment with CDDP (72 h) was significantly less toxic to sh-A1.S/sh-A2.4 cells than the control sh-luc/sh-luc and wt cells (Fig. 3E). In contrast we detected no difference in the extent of growth inhibition evoked by BTZ treatment between the examined cell sublines (Fig. 3F). In general, our results showed that neither the isoform-specific reduction of HSPA1 or HSPA2 expression nor double knockdown of HSPA1 and HSPA2 elevated sensitivity of NSCLC cells to BTZ. In the context of resistance to CDDP, our results showed that decreasing HSPA1 and HSPA2 levels may render NSCLC cells more resistant to the drug.

### VER-155008 (VER) and JG-98, pan-inhibitors of HSPA family proteins reduces growth of NSCLC cells

Next, we examined the impact of global HSPAs inhibition on growth and chemoresistance of NSCLC cells. For this purpose we used VER and JG-98, small molecule inhibitors. VER specifically competes with ATP for the binding to nucleotide binding domain (NBD) in HSPA1, HSPA8 and HSPA5^38^, and also with high probability to NBD of other HSPAs, including HSPA2, due to high structural similarity of their NDB domains^39^. Mechanistic studies revealed that VER binding arrests the NBD domain in a half-open conformation and thereby interrupts allosteric control between NBD and substrate binding domain^40^. JG-98, in turn, binds tightly a conserved allosteric pocket in NBD of HSPA1 and HSPA8 proteins. JG-98, similarly to VER does not distinguish between highly homologous HSPA isoforms^41^. This binding interrupts the interaction between HSPAs and BAG co-chaperones, in particular BAG3, BAG1 and BAG2 proteins^41^. Recently, HSPA-BAG3 complexes were recognized as broad-acting regulators of cancer cell signaling and suggested as a potential anticancer target^42,43^. Antiproliferative effects of VER^35,44–46^ and JG-98^41^ on some cancer cells were reported earlier.

**Figure 4:**
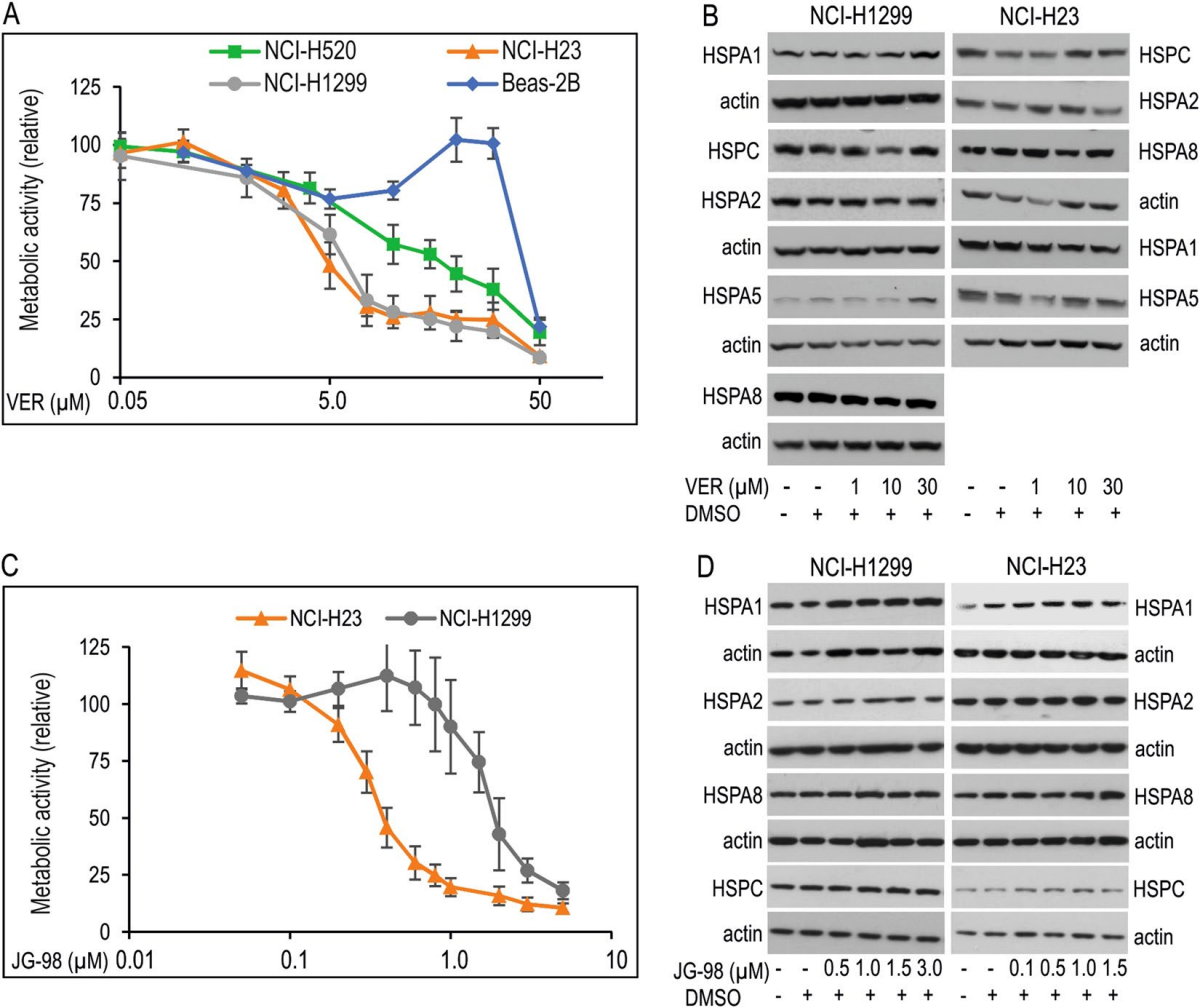
Effect of pan-HSPA inhibitors on cell viability and HSPAs expression in NSCLC cells. (**A**,**C**) Dose-response curves of VER-155008 (VER) (**A**) and JG-98 (**C**) in NSCLC cells. Cell viability was measured following 72 h treatment with VER (0–50 µM) or JG-98 (0–5 µM) using MTS assay. Results (mean ± SD from at least three independent measurements, each in three technical repeats) are expressed relatively to untreated control. (**B**,**D**) Expression of HSPA1, HSPA2, HSPA5, HSPA8 and HSPC proteins in NSCLC cell lines following 24 h treatment with VER or JG-98. Representative immunoblots are shown (n ≥ 3), actin was used as a protein loading control.

As revealed by MTS assay, VER significantly reduced viability of NSCLC cells in a dose-dependent manner (Fig. 4A). However, it was relatively safe to bronchial epithelial Beas-2B cells expressing high levels of HSPAs (Figs 1B and 4A). In cancer cells IC_50_ value of VER (Table 1) was the highest for NCI-H520 cells, which expressed HSPA1, HSPA2 and HSPA8 proteins at low levels in comparison to other NSCLC cell lines in our model (Figs 1B and 4A). For NCI-H23 and NCI-H1299 cell lines IC_50_ values of VER were comparable (Table 1). Also JG-98 effectively decreased viability of NSCLC cells (Fig. 4C). However, in contrast to VER, IC_50_ values of JG-98 were different in NCI-H23 and NCI-H1299 cells, the last ones represented the cells more resistant to the inhibitor (Table 1).

We found that HSPAs inhibition by increasing doses of JG-98 (0.5–3.0 µM) did not resulted in compensatory induction of HSPAs and HSPC expression in NCI-H23 and NCI-H1299 cells (Fig. 4D). In the case of VER, only a high dose (30 µM) stimulated expression of ER-resident HSPA5 and stress-inducible HSPA1 in NCI-H1299 cells (Fig. 4B). This suggests that VER, in contrast to JG-98, could induce the unfolded protein response and classical stress response in some NSCLC cell lines. Collectively, our results showed that simultaneous inhibition of HSPA family proteins by VER and JG-98 had strong antiproliferative effect on NSCLC.

**Figure 5:**
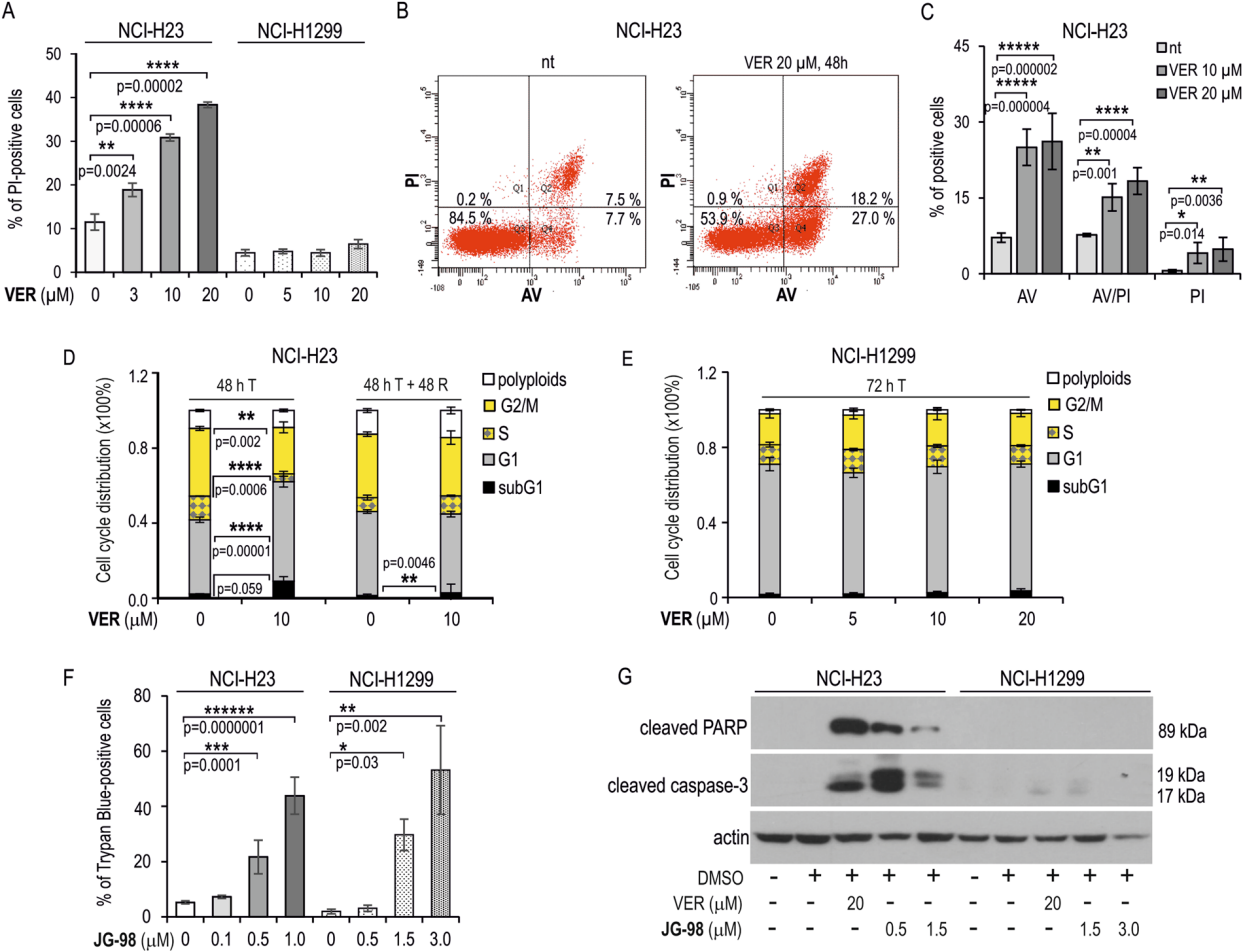
Inhibition of HSPAs by VER and JG-98 lead to apoptosis in NCI-H23 but not in NCI-H1299 cells. (**A**) Cell death detection after 72 h treatment of NCI-H23 and NCI-H1299 cells with VER-155008 (VER). Results of propidium iodide (PI) uptake test show mean values ± SD (n = 3, each in duplicate). Statistical significance was determined using two-tailed t-test. (**B**,**C**) Flow cytometry analysis of cell death in NCI-H23 cells incubated with VER performed using Annexin V (AV)/PI double labeling method. Cells were plated onto 6-well dishes (2 × 10^4^/well) and treated for 48 h. Graph shows percentage of cells labeled with AV and/or PI (mean value ± SD, n = 3). (**D**,**E**) Distribution of the cell cycle phases in NCI-H23 (**D**) and NCI-H1299 (**E**) cells non-treated (0) or treated for 48 h with VER (5, 10, 20). Cell cycle phases distribution was analyzed immediately after treatment (72 h T) or followed by 48 h of recovery (48 T + 48 R). Graph shows percentage of cells (mean ± SD) calculated from seven (NCI-H1299 cells) or two (NCI-H23) independent experiments, each in duplicate. Statistical significance was calculated using two-tailed t-test. **(F)** Cell death detection after 72 h treatment of NCI-H23 and NCI-H1299 cells with JG-98. Results of trypan blue staining assay show mean values ± SD (n = 3, each in duplicate). (**G**) Western blot analysis of activated caspase-3 and cleaved PARP in cells non-treated and treated with JG-98 or VER (72 h). Representative immunoblots are shown (n = 2), actin was used as a protein loading control. In (**A**), (**C**) and (**F**) statistical significance was determined by one-way analysis of variance (ANOVA) and Scheffe’s pairwise post hoc test.

Previous studies showed that VER potently induced apoptosis in various types of cancer cells^35,38,47,48^, but it has not been shown if JG-98 can activate apoptosis in cancer cells. We found here that both inhibitors can induce apoptosis in NSCLC albeit the effect can be cell-type dependent. Treatment with VER caused a massive induction of cell death in NCI-H23, but not in NCI-H1299 cells (Fig. 5A). Flow cytometry analysis of NCI-H23 cells confirmed significant increase in a fraction of annexin V (AV)-positive and AV/PI double positive cells following 48 h treatment with VER (10 or 20 µM) when compared to non-treated control (Fig. 5B,C). This indicates that VER can activate apoptosis only in NCI-H23 cells. The analysis of the cell-cycle phase distribution in NCI-H23 cells exposed to VER (10 µM, 48 h) revealed that apoptosis induction paralleled G1 phase cell cycle arrest with concomitant reduction in G2/M and S fractions in relation to the untreated cells (Fig. 5D). However, the cell cycle block was released within 48 h after VER withdrawal (Fig. 5D). In contrast, VER (0–20 µM) had no effect on the cell-cycle in NCI-H1299 cells (Fig. 5E).

Because JG-98 is a fluorophore having a broad fluorescence emission spectrum (Fig. S2), in order to characterize its cytotoxic effects we used non-fluorescent methods for cell death detection (trypan blue staining and Western blot analysis of apoptotic markers expression). Treatment of NCI-H23 and NCI-H1299 with increasing doses of JG-98 resulted in significant increase in the number of dead cells (Fig. 5F). This response to JG-98, similarly as for VER, was accompanied by the presence of cleaved caspase-3 and cleaved PARP in NCI-H23 cells, but not in NCI-H1299 cells (Fig. 5G). Our findings indicate that JG-98 is cytotoxic for NSCLC cells and, depending on cellular context, it can induce apoptotic or non-apoptotic cell death. Due to physical properties of JG-98 molecule we omitted the analysis of the cell cycle distribution in inhibitor-treated cells.

JG-98 disrupts HSPAs interaction with the co-chaperone BAG3 and affects signaling pathways important for cancer development. Here we observed that also treatment of NCI-H1299 and NCI-H23 with VER resulted in substantial decrease in expression of BAG1 and nuclear variant BAG1L. Simultaneously, an increase in BAG3 level was observed in NCI-H1299 cells incubated with a high dose of VER (Fig. S3). These observations suggest that antiproliferative action of VER could also be related to alterations in the equilibrium of BAG proteins.

### Combination of pan-HSPA inhibitors with BTZ increases toxic effect on NSCLC cells

**Figure 6:**
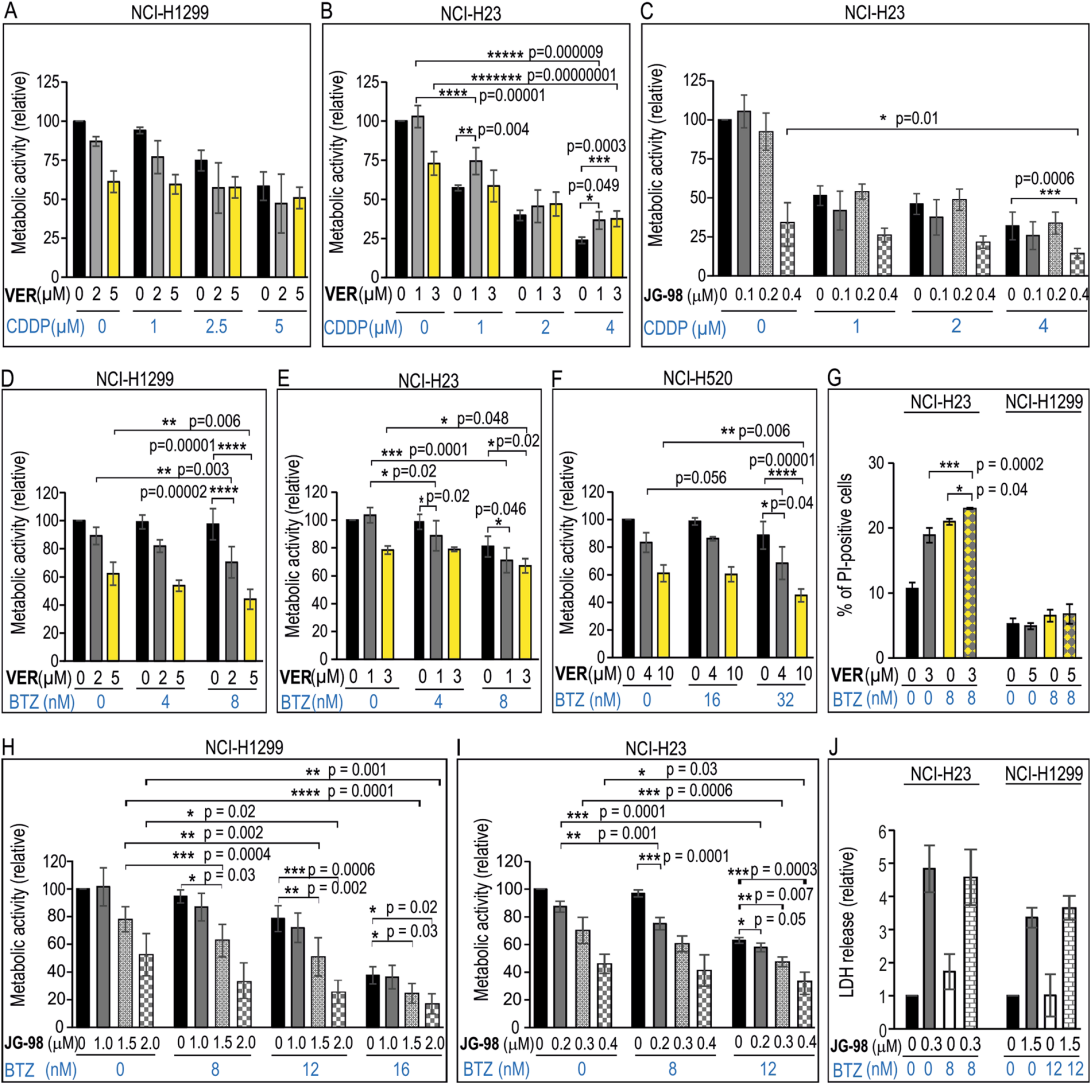
Viability of NSCLC cells after single or combined treatment (72 h) with (**A**,**B**) VER-155008 (VER) and cisplatin (CDDP); (**C**) JG-98 and CDDP; (**D**–**G**) VER and bortezomib (BTZ); and (**H**,**I**) JG-98 and BTZ. Cell viability was measured using MTS assay. Results are expressed in relation to the untreated control (mean ± SD, n ≥ 3, each in triplicate). (**G**,**J**) Detection of dead cells following 72 h treatment of cells with VER (G), JG-98 (J), BTZ or both drugs. In (**G**) results of propidium iodide (PI) uptake test show mean values ± SD (n = 4, each in duplicate). In (**J**) results of lactate dehydrogenase (LDH) release assay are shown (mean values ± SD, n ≥ 4, each in duplicate). Statistical significance was determined using two-tailed t-test (**A**–**C**) or one-way analysis of variance (ANOVA) with Scheffe’s (**D**–**G**) or Duncan’s (**H**,**I**) adjustment for pairwise comparisons.

Having established that HSPAs inhibition reduced viability of NSCLC cells, it was important to examine whether combination of pan-HSPA inhibitors with either platinum derivatives or BTZ would have higher antiproliferative activity. In our experiments we used the drugs at concentration corresponding to IC_50_ values or lower. Using MTS assay we found that combination of VER with CDDP or CPT did not reduced viability of NCI-H1299, NCI-H358 and NCI-H520 cell lines to a significantly greater extent than treatment of cells separately either with the inhibitor or platinum derivatives (Figs 6A, S4). Moreover, the combination of VER and CDDP was even less toxic to NCI-H23 cells than a single treatment with CDDP (Fig. 6B). This confirmed that decreasing HSPA1 and/or HSPA2 levels in NSCLC cells may even reduce cytotoxic effect of CDDP. However, combination of JG-98 and CDDP had opposite effect to that observed in cells exposed to combination of VER and CDDP (Fig. 6B,C). We found that high doses of both CDDP and JG-98 applied together were slightly higher toxic than each compound used separately (Fig. 6C). Altogether, we showed that combining CDDP with various HSPA inhibitors have different effect on cell viability.

In contrast, combination of BTZ with VER had significantly stronger antiproliferative effect on NCI-H1299 (Fig. 6D), NCI-H23 (Fig. 6E) and NCI-H520 cells (Fig. 6F) than a single drug treatment. Results of PI uptake assay showed that BTZ alone, similarly to VER alone, evoked significant cytotoxic effect on NCI-H23 cells, but not on NCI-H1299 cells (Fig. 6G). Also, in comparison with a single drug treatment, combination of VER and BZT significantly increased a number of PI-positive cells in NCI-H23 cells, but not in NCI-H1299 (Fig. 6G). This finding indicates that combination of VER with BZT could have cytotoxic or cytostatic effect depending on a cell line/cellular context.

Using MTS test we showed that the combination of BTZ with JG-98 had significantly stronger antiproliferative effect on NCI-H1299 (Fig. 6H) and NCI-H23 (Fig. 6I) cells than a compound used alone. However, results of lactate dehydrogenase (LDH) cytotoxicity assay, which measures the release of LDH from damaged cells, revealed similar cytotoxicity of combination of BTZ with JG-98 on NCI-H23 and NCI-H1299 cells to that observed after single drug treatment (Fig. 6J). This finding indicate greater potential of JG-98 to induce NSCLC cell death comparing to VER.

## Discussion

In this work we showed that simultaneous inhibition of HSPA isoforms (Fig. 4B), in contrast to both single paralog-specific (Figs 2C–F, S1C–F) and double knockdown (Fig. 3B–D) of HSPA1 and HSPA2 isoforms, evoked significant antiproliferative effect on human NSCLC cells. We found that the anticancer activity of pan-HSPA inhibition is potentiated by proteasome inhibitor BTZ (Fig. 6D–J) but not by platinum derivatives (Fig. 6A–C, S4). Of note, neither single nor double depletion of HSPA1 and HSPA2 sensitized NSCLC cells to anticancer drugs used in this study (Figs 2G–K and 3E,F, S1G–K).

Results of multiple studies focused on assessing the potential dependence of cell proliferation on HSPA1 expression^40,49–54^, although are not fully consistent, suggest a few important conclusions. It seems clear that HSPA1 does not support growth and propagation of non-tumorogenic cells^49,50,52,54^. Also, it turned out that the use of adenoviral vectors to deliver antisense HSP70 cDNA can affect viability of cancer cells in HSP70-independent way^51,54^. When it comes to the effect of HSPA1 on cancer cell growth, some studies indicated that this protein is critical for proliferation^50,52^, while other negate such a dependence^37,51^. Another studies demonstrated that only simultaneous depletion of HSPA1 and HSPA8 can effectively block cancer cell proliferation^40,53^. In turn, the acknowledged growth-promoting role of HSPA2 in cancer cells has not been questioned so far^50,55,56^.

Our results show that neither HSPA1 nor HSPA2 isoforms, separately or together, support proliferation of NSCLC cells (Figs 2C–F and 3B–D, S1C–F). Thus, our results are consistent with data published by Endo *et al*.^37^, who showed that siRNA-mediated silencing of HSPA1 expression did not affect viability of A459 cells. We found that proliferation of NSCLC cells was blocked only if cells were treated with pan-HSPA inhibitors (Figs 4A,C and 5D). In this respect, our results are consistent with reports showing that several HSPAs co-operate in sustaining growth of cancer cells other than NSCLC^40,53^. Thus, it seems reasonable to assume that the degree of reliance on HSPA1 or HSPA2 for cell proliferation can differ in cancer cells of diverse histological origin and/or depending on cellular context. This also indicates, that members of the HSPA family appear to co-operate as a group of redundant, growth-promoting factors in NSCLC cells. Of note, we found that immortal bronchial epithelial Beas-2B cells, in spite of containing high levels of HSPA paralogs, were insensitive to VER-induced proliferation arrest (Fig. 4A), thus their growth seems relatively independent on HSPAs expression.

Our study is the first one undertaken to compare the effect of depletion of HSPA1 and/or HSPA2 on sensitivity of NSCLC cells to platinum derivatives and BTZ. We found no relationship between the endogenous levels of HSPA2 or HSPA1 (Fig. 1B) and susceptibility of NSCLC cells to these drugs (Fig. 1A,C). Also, both single and double knockdown of *HSPA1* and/or *HSPA2* genes did not increase sensitivity of NSCLC cells to the drugs (Figs 2G–K, 3E,F and S1G–K). One of the most interesting observations we made is that simultaneous inhibition of HSPA isoforms by the pan-HSPA inhibitors (VER, JG-98) sensitized NSCLC cells to BTZ (Fig. 6D–I), but not to CDDP (Figs 6A,B and S4A,B) or CPT (Fig. S1C,D). Our observation suggests that HSPAs form a highly redundant network of chaperones in NSCLC cells that might counteract the BTZ-induced proteotoxic stress. Results showing that pan-HSPA inhibition synergizes with proteasome arrest in breast cancer or multiple myeloma cells were also provided by others^41,57^. Accordingly, the concomitant inhibition of multiple HSPAs and proteasome function may represent a potent innovative approach to anticancer therapy.

We also observed that both decreasing the levels of HSPA1 and HSPA2 (separately, Fig. 2G–I; or in combination, Fig. 3E), as well as blocking HSPAs activity by VER (Figs 6B, S1C,D) did not alter NSCLC cells sensitivity to CPT but instead made them more resistant to CDDP. Our results contradict previous finding showing that deficit of HSPA1 in NSCLC cells (A549 cell line) increased sensitivity to CDDP^37^. The discrepancy can be explained either by cell-type specific effects or by potential influence of methodological issues. In our study we used cell lines stably expressing shRNA, whereas Endo *et al*.^37^ used Lipofectamine 2000-mediated transport of siRNA. Earlier, it has been shown that the liposome-based carriers can activate expression of the genes encoding HSPA1 protein as well as numerous genes involved in the cell cycle control and pro-apoptotic pathways^58^.

Our general finding that reduction in HSPAs activity in NSCLC cells did not sensitize them to CDDP is not surprising. There are various mechanisms of resistance of cancer cells to CDDP, both genetic and epigenetic, that had been classified as pre-target, on-target, post-target, and off-target^59^. In our recent review (Krawczyk *et al*., 2018) we underlined that there is no universal correlation between endogenous HSPs expression and the CDDP resistance. If the above-mentioned relationship is observed, it mostly appears as a cancer cell type-related phenomenon and its manifestation depends on combination of multiple factors including tumor microenvironment^10^. In the context of potential impact of HSPAs on resistance of NSCLC cells to CDDP it is worth noting that combination of CDDP with VER or JG-98 may evoke opposed effect on NSCLC viability. Comparing to results of a single drug treatment, CDDP combined with VER showed decreased toxicity (Fig. 6B), but its combination with a high dose of JG-98 was more toxic to the cells (Fig. 6C). It seems that various effects of these two pan-HSPA inhibitors on antiproliferative action of CDDP can be explained among others by different mode of action of VER and JG-98^38,40,41^ or can represent their off-target interactions. In this point it should be kept in mind that NCI-H1299 cells deficient in HSPA1 and/or HSPA2 also showed reduced sensitivity to CDDP (Figs 2G,H and 3E).

One of the effects associated with the use of anticancer cytostatic agents may be the stimulation of the stress response manifested by high induction of HSP genes expression, in particular the inducible *HSPA1A/1B* genes. Such phenomenon has been observed in cancer cells treated with HSPC inhibitors^60^, BTZ^61^, and occasionally with CDDP^10^. Up-regulation of cytoprotective, anti-apoptotic HSPs in response to chemotherapeutic drugs is considered an adverse effect, potentially limiting the effectiveness of chemotherapy. Although this response has not been systematically studied, it seems noteworthy that inhibition of HSPA by 2-phenylethynesulfonamide (PES, pifithrin-µ) increased the toxic effect of CDDP (and also docetaxel and gemcitabine), in combination with HSPC inhibitor 17-AGG in bladder cancer cells^62^. Similarly shRNA-mediated depletion of HSPA1 increased toxicity of BTZ in bladder cancer cells^61^, and the combination of HSPAs inhibitor JG-98 and proteasome inhibitor MG-132 has synergistic effect in breast cancer cells^41^.

In our study we observed that CDDP (Fig. 1D) and BTZ (Fig. 1E) exerted an opposite and cell-type dependent changes in HSPA1 and HSPA2 expression. CDDP did selectively upregulate expression of HSPA2. Taking into account, that depending on cell type and environmental conditions exposure of cancer cells to CDDP may affect HSPA1 expression in a highly variable manner, a constant level of HSPA1 in CDDP-treated NSCLC cells is not an unusual observation^10^. Similar insensitivity of the *HSPA1* gene to CDDP in NSCLC cells (A549 cell line) was reported earlier^63^. Although NSCLC cells responded to BTZ by increasing the level of HSPA1 (Fig. 1E), we showed that this effect should not simply be considered an adaptive response to BTZ-induced proteotoxic stress, since neither selective HSPA1 knockdown, nor double HSPA1 and HSPA2 knockdown sensitized cells to BTZ (Figs 2J,K, 3F and S1J,K). In fact, the biological meaning of BTZ-induced accumulation of HSPA1 for the fate of NSCLC cells is not known. Similarly, at present it remains unclear what is the biological role for HSPA2 accumulation in NSCLC cells exposed to CDDP, as well as for massive fall in the level of HSPA2 upon BTZ treatment.

Our findings that pan-HSPA inhibition effectively reduced viability of NSCLC cells raised the question on cell death pathways triggering under conditions of impaired chaperones activity. As we observed, both VER and JG-98 can trigger apoptosis (Fig. 5B,C,G) but only in an apoptosis-sensitive NSCLC cell line. Moreover, apoptosis-resistant NCI-H1299 cells, which are both deficient in p53 protein and defective in the executive phase of extrinsic death receptor apoptosis pathway^64^, showed different response to HSPAs inhibition by VER and JG-98 (Fig. 5A,F). Since VER caused cytostatic effect (Fig. 5A), JG-98 massively induced non-apoptotic cell death (Fig. 5F). Such a distinct response of NCI-H1299 cells to VER and JG-98 may be at least partially explained by our finding that VER, but not JG-98 stimulated production of HSPA1 and HSPA5 pro-survival proteins (Fig. 4B,D). NSCLC cells in response to VER also showed alteration in the levels of BAG1 and BAG3 (Fig. S3), proteins having potent anti-apoptotic activity^65,66^. Altogether, increased levels of these anti-apoptotic proteins upon VER treatment can be regarded a compensatory response capable to alleviate death-inducing stimuli upon global HSPAs inhibition. Interestingly, such a response was not activated by JG-98 what coincided with the activation of cell death in NCI-H1299 cells.

Taking into consideration that VER and JG-98 interfere with HSPA activity via different mechanism, our results suggest that blocking HSPA interaction with a network of co-chaperones can effectively disrupt pro-survival signaling in NSCLC cells, even these ones resistant to apoptosis. JG-98, a novel class of allosteric inhibitors of HSPAs, allows for effective disruption of HSPAs-BAG3 interaction^43^. Thus, our findings support earlier conclusions that HSPA-BAG3 complexes can be considered as broad-acting regulators of cancer cell signaling and a promising anticancer target^42^. Our results may also be beneficial to further develop novel HSPA inhibitors for clinical application.

To sum up, results of our study underline the complexity of response of cancer cells to cytostatics as well as a complex role of HSPs in tumor propagation and response to chemotherapy. Our results show that simultaneous inhibition of HSPA family members has potent anticancer activity and offers the potential for therapeutic selectivity, therefore could be regarded as promising future anticancer strategy for NSCLC.

## Materials and Methods

### Cell culture and experimental conditions

BEAS-2B (virus-transformed non-tumorigenic bronchial epithelial cells; CRL-9609) and non-small lung cell cancer (NSCLC) cell lines: NCI-H1299 (non-small cell lung carcinoma, CRL-5803), NCI-H23 (adenocarcinoma, CRL-5800), NCI-H520 (squamous cell carcinoma, HTB-182), and NCI-H358 (bronchioalveolar carcinoma, CRL-5807) cells were purchased from ATCC (Manassas, VA, USA). Cells were cultured at 37 °C under standard conditions (5% CO_2_, 95% humidity, 21% O_2_ concentration). BEAS-2B cells were grown in serum-free bronchial epithelial growth medium (Lonza Ltd, Basel, Switzerland), not allowing cells to exceed 70% confluence. NSCLC cell lines were cultured in RPMI (Sigma-Aldrich) supplemented with 10% heat-inactivated fetal bovine serum and antibiotics (gentamycin or penicillin-streptomycin). All cells were regularly checked for mycoplasma contamination.

### Incubation experiments

The following stock solutions were used: VER-155008 (VER) (20 mM in DMSO; Sigma–Aldrich), JG-98 (1.5 mM in DMSO; MedChemExpress, NJ, USA), bortezomib (BTZ) (1.6 mM in DMSO; Selleckchem, Houston, TX, USA); cisplatin (CDDP) (1 mg/ml, for infusion), carboplatin (CPT) (10 mg/ml, for infusion). Working solutions were prepared fresh before each experiment in culture medium (without antibiotics). Cells were incubated with: 0–50 µM VER, 0–5 µM JG-98, 0–256 nM BTZ, 0–100 µM CDDP, 0–200 µM CPT. Control cells were incubated with medium containing DMSO. The effect of drug treatment on cell viability was determined using CellTiter 96 Aqueous One Solution Assay according to a manufacturer’s protocol (Promega; Madison, WI, USA). Cells (3 × 10^3^, NCI-H1299; 5 × 10^3^, NCI-H23; 5 × 10^3^, NCI-H358; 15 × 10^3^, NCI-H520; 5 × 10^3^, Beas-2B; cells per well) were plated into 96-well plates and incubated with chemicals for 72 hours. The absorbance of formazan product was measured (λ = 490 nm) using microplate reader. Absolute IC_50_ values (mean ± 95% confidence intervals) for CDDP, BTZ, VER and JG-98 were calculated by fitting the dose-response curve using GraphPad Prism Software (GraphPad Software; La Jolla, CA).

### Protein extraction and western blot analysis

Cells were seeded in 6-cm dishes with a maximum confluency of 50–70% and 24 h after plating were exposed to the drugs or inhibitor for 24 h. To prepare total protein extracts, cells were lysed by scrapping in IP buffer (50 mM Tris–HCl (pH 7.5), 150 mM NaCl, 0.1% Nonidet P-40, 50 mM NaF, 1 mM DTT, 1 mM PMSF) supplemented with Phosphatase Inhibitor cocktail 2 and protease inhibitor mixture. After incubation on ice (20 min), lysates were centrifuged (4 °C for 15 min at 22.000 × *g*). Total protein content was determined using Protein Assay Kit (Bio-Rad; Hercules, CA). 25–35 μg of total proteins were fractionated by SDS-PAGE on 8% polyacrylamide gels and transferred on nitrocellulose membrane using Trans Blot Turbo system (Bio-Rad) for 10 min. Membrane was blocked (60 min) in 5% nonfat milk/TTBS (0.25 M Tris–HCl (pH 7.5), 0.15 M NaCl, and 0.1% Tween-20), and incubated (overnight at 4 °C or 1 h at 37 °C) with primary antibodies (Table 3). Antibody-antigen interaction was detected using secondary antibody and visualized using SuperSignal® West Pico Chemiluminescent Substrate Kits (Pierce) or Clarity ECL Western Blot Substrate (Bio-Rad; Hercules, CA). Immunodetection of β-actin was used as a loading control. Relative expression was calculated using ImageJ Software^67^.

**Table 3:** List of antibodies used in Western blot analyses.

	Host/clonality	Clone	Catalog numer/RRID	Source	Dilution
**Primary**					
HSPA1	Mo/M	C92F3A-5	ADI-SPA-810-F/AB_311860	Enzo, Life Sciences, Famingdale, NY	1:5000
HSPA2	Ra/M	EPR4596	Ab108416/AB_10862351	Abcam, Cambridge, UK	1:5000
HSPA5	Mo/M	A-10	Sc-376768/nd	Santa Cruz Biotechnology, Inc., Dallas, USA	1:1000
HSPA8	Mo/M	B-6	Sc-7298/AB_627761	Santa Cruz Biotechnology, Inc., Dallas, USA	1:7500
HSPC	Mo/M	AC88	ADI-SPA-830-F/AB_11181197	Enzo, Life Sciences, Famingdale, NY	1:1000
BAG1	Mo/M	19	611868/AB_399348	BD Biosciences, San Diego, CA	1:1000
BAG3	Mo/M	19	Ssc-136467/AB_10647772	Santa Cruz Biotechnology, Inc., Dallas, USA	1:1000
cleaved caspase-3	Ra/M	5A1E	9664/AB_2070042	Cell Signaling Technology,	1:2000
cleaved PARP	Ra/M	D64E10	5625/AB_10699459	Cell Signaling Technology,	1:2000
β-actin (HRP)	Mo/M	AC15	A3854/AB_262011	Merck KGaA, Darmstadt, Germany	1:20000
**Secondary**					
Anti-Mo IgG (HRP)	Go		AP124P/AB_90456	Millipore, Billerica, MA	1:5000
Anti-Ra IgG (HRP)	Go		AP132P/AB_90264	Millipore, Billerica, MA	1:2000

Abbreviations: RRID, Research Resource Identifier; WB, Western Blot; M, monoclonal; Go, goat; Mo, mouse; nd, no data; Ra, Rabbit; HRP, horseradish peroxidase.

### Generation of lentiviral shRNA vectors

The control non-targeting shRNA vector and lentiviral shRNA vectors targeting coding sequence of human *HSPA2* (Entrez Gene: 3306) and *HSPA1A/HSPA1B* (Entrez Gene: 3303/4) genes were constructed as described before^68^, by insertion of appropriate double-stranded oligonucleotides into the pLVX-shRNA1 or pLVX-shRNA2 vector (Clontech, Takara Bio, Mountain View, CA, USA). The later vector, coding for ZsGreen1 green fluorescent protein, was used for generation of double HSPA1 and HSPA2 gene knockdown cell lines. To target *HSPA1A/B* genes the following shRNA were selected: shRNA-A1.S according to Yaglom *et al*.^52^, and shRNA-A1.N designed and characterized by us. The target shRNA sequences are collected in Table 1. Infectious lentiviruses were generated by transfecting shRNA-encoding plasmids into HEK293T packaging cells according to the manufacturer’s instructions (Clontech/Takara Bio, Lenti-X shRNA Expression System). A viral titer > 5 × 10^5^ IFU/ml in virus-containing supernatants was confirmed using Lenti-X GoStix (Clontech/Takara Bio). Cells were transduced with supernatants containing lentiviruses for 24 h at 37 °C with addition of polybrene (4 µg/ml for NCI-H1299 and Beas-2B; 8 µg/ml for NCI-H23, NCI-H358 and NCI-H520 cells). A similar number of cells infected with non-targeting or targeting lentiviral vectors remained viable after transduction and survived puromycin (1 μg/ml for NCI-H1299 or 2.5 μg/ml for NCI-H23 cells) selection for establishment of stably transduced cell lines. Combined knockdown of HSPA1 and HSPA2 isoforms was achieved by transduction of cells with lentiviruses bearing shRNA-A2.4 sequence in pLVX-shRNA2 vector followed by fluorescence activated cell sorting of live ZsGreen-positive cells. Then the cells were transduced with lentiviruses bearing shRNA-A1.S sequence in pLVX-shRNA1 vector and subjected to puromycin selection. The control cell line was generated by subsequent transductions of cells with pLVX-shRNA2 and pLVX-shRNA1 vectors encoding shRNA-luc sequence.

### Cell proliferation and clonogenic assay

NCI-H1299 (2 × 10^3^/well) and NCI-H23 (2.5 × 10^3^/well) were seeded onto 96-well plates. At the indicated times (24, 48, 72 and 96 h after plating) the metabolic activity of cells was measured as described above. The metabolic activity values were calculated relative to the readouts obtained after 24 h of cell growth. For crystal violet proliferation assay cells at the indicated time after plating were washed with PBS, fixed in ice-cold methanol, stained with 0.1% crystal violet for 30 min, rinsed extensively with distilled water, and dried. Cell-associated dye was extracted with 10% acetic acid, aliquoted (200 μl) and the absorbance was measured at 595 nm using microplate reader.

#### Clonogenic assay

NCI-H1299 (1 × 10^3^/well) and NCI-H23 (2.5 × 10^3^/well) cells were plated onto 6-well dishes, cultured for 6–7 (NCI-H1299) or 10–11 (NCI-H23) days in standard growth medium, fixed with methanol (−20 °C), dried, and dyed with crystal violet. Numbers of colonies were counted manually.

### Analyses of cell death and cell-cycle distribution

#### Propidium iodide (PI) uptake test

Cells (NCI-H23, NCI-H1299; 1.5 × 10^4^/well) were plated on 6-well dishes, cultured for 24 h and exposed to VER alone or in combination with BTZ for up to 72 h. Next, both adherent and floating cells were collected by trypsin digestion, rinsed with PBS, stained with PI (1 µg/ml, 10 min, Sigma Aldrich), and analyzed by flow cytometry.

#### Trypan Blue staining assay

Cells (NCI-H23, 2 × 10^4^/well; NCI-H1299, 1 × 10^4^/well) were plated into 6-well dishes, cultured for 24 h, and exposed to JG-98 for 72 h. The cytotoxic effect was analyzed by staining of both adherent and floating cells with 0.4% Trypan Blue dye (Bio-Rad; Hercules, CA, USA). Cells were harvested by trypsinization, mixed 1:1 (v/v) with the dye solution and incubated for few minutes, then cytospined (1 min, 1000 RPM, Thermo Shandon Cytospin 3, Thermo Shandon Ltd., Runcorn, UK) onto glass slides. Cells (n = 1000) were counted manually under a light microscope in randomly-chosen fields. The unstained cells were considered viable, blue-stained cells were considered dead.

#### Annexin V (AV)/PI staining

NCI-H23 (2 × 10^4^/well) cells were seeded on 6-well plates, the next day cells were incubated with VER for 48 h. Cells, both floating and adherent, were harvested by trypsynization and stained using eBioscience Annexin V Apoptosis Detection Kit PE (Invitrogen; Life Technologies; Carlsbad, CA) according to the manufacturer’s instructions. Fluorescence was analyzed using FACSCanto flow cytometer (Becton Dickinson; San Jose, CA, USA NJ). A blue laser was used for excitation (488 nm) and the FITC (530/30 nm) and PE (575/26 nm) channels for the detection of AV- and PI-stained cell, respectively.

#### LDH release assay

Cells (NCI-H1299, 3 × 10^3^/well; NCI-H23, 5 × 10^3^/well) were plated into 96- well plates and incubated with BTZ and/or JG-98 for 72 hours. The cytotoxic effect of the drug(s) was measured using CytoTox 96 Non-Radioactive Cytotoxicity Assay according to a manufacturer’s protocol (Promega; Madison, WI, USA).

#### Analysis of the cell-cycle distribution

Cells (NCI-H23, 2 × 10^4^/well; NCI-H1299, 1 × 10^4^/well) were plated into 6-well plates and incubated for 48 h (NCI-H23) or 72 h (NCI-H1299) with VER. At the indicated time cells were harvested by trypsinzation, rinsed with PBS, fixed with ice-cold 70% ethanol, digested with RNase (100 µg/ml) and stained with 100 µg/ml PI solution. DNA content was analyzed to monitor the cell cycle changes.

### Statistical analysis

Unless otherwise stated, all data were shown as mean ± standard deviation of the mean (SD). Univariate statistical significance was determined by one-way analysis of variance (ANOVA) with Scheffe’s or Duncan’s adjustment for pairwise comparisons. Difference significance between two groups was determined by t-test for independent samples. The p-value of less than 0.05 was considered statistically significant. STATISTICA 12 (StatSoft, Tulsa, OK, USA) was used for the analyses.

## Supplementary information

Supplementary Information

## Data Availability

All materials and data are available upon reasonable request to the corresponding author.
