# Supplementary material for: Functional redundancy of HSPA1, HSPA2 and other HSPA proteins in non-small cell lung carcinoma (NSCLC); an implication for NSCLC treatment
Source: Sci Rep. 2019 Oct 7;9:14394. doi: 10.1038/s41598-019-50840-7 (PMC6779912; doi:10.1038/s41598-019-50840-7)
Supplement: Supplementary file 1 — Supplementary Information [file 41598_2019_50840_MOESM1_ESM.pdf]

Supplementary Information

Functional redundancy of HSPA1, HSPA2 and other HSPA proteins in non-small cell lung carcinoma (NSCLC); an implication for NSCLC treatment

Damian Robert Sojka[1], Agnieszka Gogler-Pigłowska[1], Natalia Vydra[1], Alexander Jorge Cortez[1], Piotr Teodor Filipczak[1,2], Zdzisław Krawczyk[1], Dorota Scieglinska[1]*

[1] Center for Translational Research and Molecular Biology of Cancer, Maria Sklodowska-Curie Institute – Oncology Center, Gliwice Branch, 44-101 Gliwice, Poland

[2] Lung Cancer Program, Lovelace Respiratory Research Institute, Albuquerque, New Mexico 87108, USA

Corresponding author

*Dorota Scieglinska, dorota.scieglinska@io.gliwice.pl, tel: (48) 32 27 89 679, fax: (48) 32 231 35 12

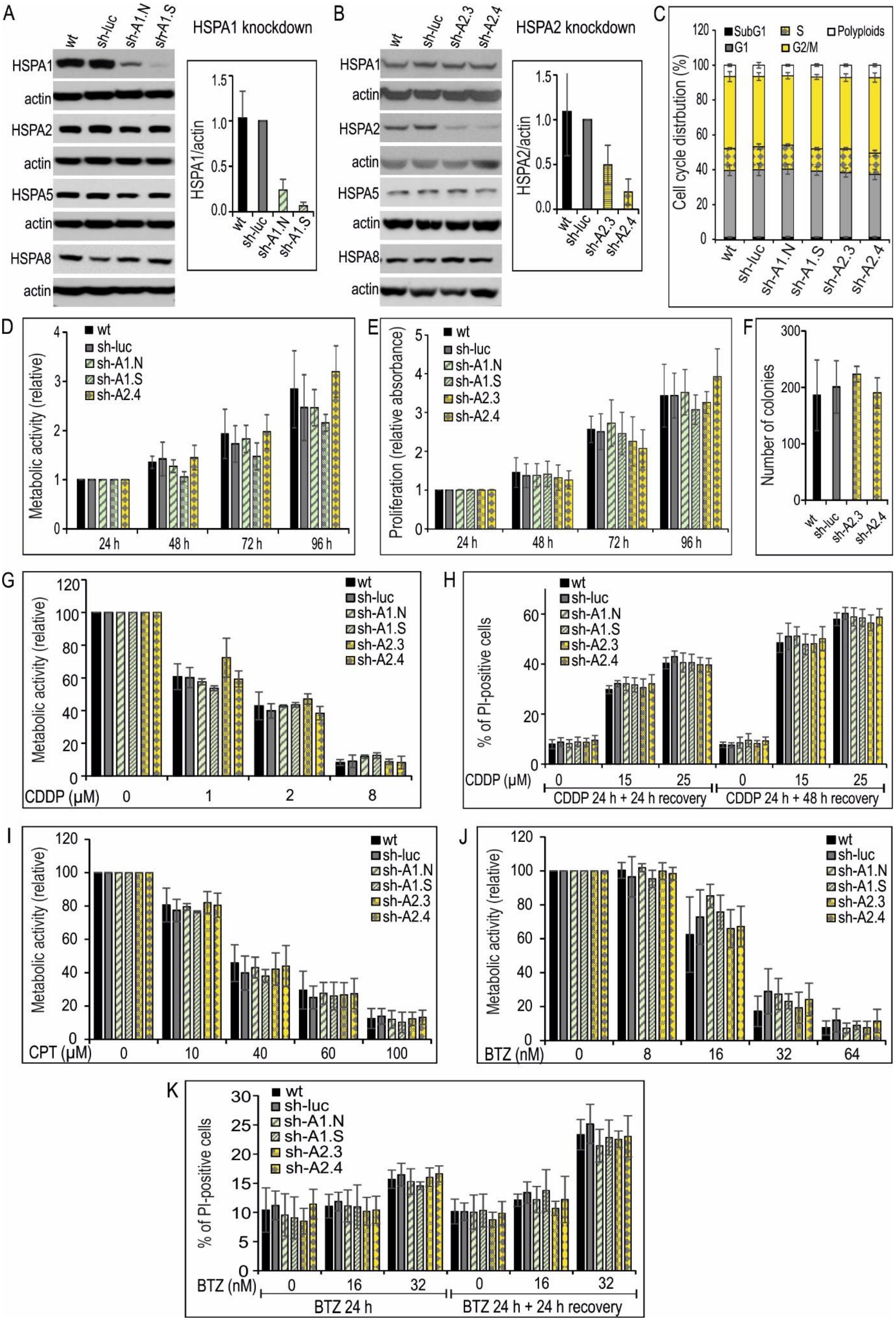

A
wt
sh-luc
sh-A1.N
sh-A1.S
HSPA1
actin
HSPA2
actin
HSPA5
actin
HSPA8
actin
HSPA1 knockdown
HSPA1/actin
B
sh-A2.3
sh-A2.4
HSPA2 knockdown
HSPA2/actin
C
SubG1
G1
S
G2/M
Polyploids
Cell cycle distrbution (%)
D
Metabolic activity (relative)
24 h
48 h
72 h
96 h
E
Proliferation (relative absorbance)
F
Number of colonies
G
CDDP (µM)
H
% of PI-positive cells
CDDP 24 h + 24 h recovery
CDDP 24 h + 48 h recovery
I
CPT (µM)
J
BTZ (nM)
K
BTZ 24 h
BTZ 24 h + 24 h recovery

Figure S1. Effects of HSPA1 or HSPA2 depletion on the proliferation, clone-forming ability and chemoresistance of NCI-H23 cells. (A-B) Silencing of *HSPA1* gene (a) or *HSPA2* gene (b) expression. Levels of HSPA family proteins in wild-type (wt); control sh-luc cells stably transduced with a non-targeting shRNA-luc sequence; sh-A1.N and sh-A1.S cell lines stably transduced with HSPA1-targeting sh-RNA-A1.N or sh-RNA-A1.S sequences, respectively (a); sh-A2.3 and sh-A2.4 cell lines stably transduced with HSPA2-targeting sh-RNA-A2.3 or sh-RNA-A2.4 sequences, respectively (b). Representative immunoblots are shown (n=3), actin is used as a protein loading control. Graph shows results of densitometric analysis of HSPA1 (a) or HSPA2 (b) proteins on Western blot (HSPA1 n=3, HSPA2 n=8). **(C)** Cell cycle phase distribution in sub-confluent cells culture at 48 after plating. Graph shows percentage of cells (mean ± SD, n = 4, each experiment in two technical replicas). (**D**) Cell proliferation evaluated using MTS assay. Results are expressed as mean ± SD (n=3, each in three technical replicas) in relation to values obtained at 24 h after cells plating. (**E**) Cell proliferation assessed by crystal violet staining assay (n = 3, each in six technical replicas). Relative absorbance of stained cells was plotted against time (24 h – 96 hours (h) after seeding) of continuous growth. **(F)** Clonogenic potential of cells plated onto 6-well dishes (2×10^3 cells / well), and cultured for 10 – 12 days. Colonies were counted manually (mean ± SD, n =5, each in three technical replicas). **(G-K)** Effects of HSPA1 or HSPA2 depletion on resistance to cisplatin (CDDP) (G-H), carboplatin (CPT) (I) or bortezomib (BTZ) (J-K). Cell viability measured using MTS assay following 72 h treatment with the drugs (G, I, J). Results are expressed relative to the untreated control (mean ± SD from at least three independent experiments, each in triplicate. Cell death detection using propidium iodide (PI) uptake test after 24 h treatment with CDDP (H) or BTZ (K) and after additional 24 or 48 h of growth without drugs. Results show mean values ± SD from three independent repeats, each in duplicate. Statistical significance was determined using two-tailed t-test.

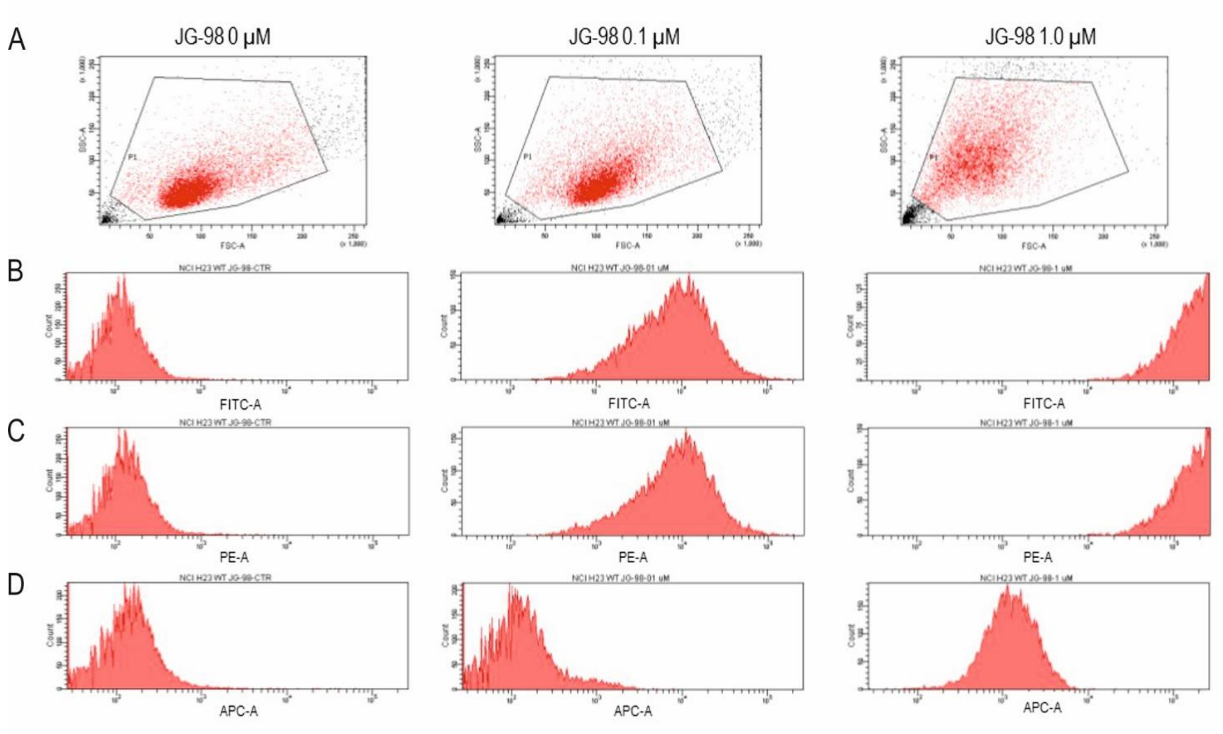

Figure S2. Fluorescence of JG-98-treated NCI-H23 cells. A) Dot-plots showing size (forward scatter, FSC) and granularity (side scatter, SCC) of untreated cells (JG-98 0 µM) or treated with JG-98 at concentration of 0.1 or 1.0 µM for 72 hours. **B-C)** Histograms showing distribution of cell fluorescence after JG-98 treatment (0, 0.1, 1.0 µM) measured using fluorescence activated cell sorting in **B)** FITC channel (excitation 488 nm, emission 530/30) **C)** PE channel (excitation 488 nm, emission 585/42) **D)** APC channel (excitation 633 nm, emission 660/20).

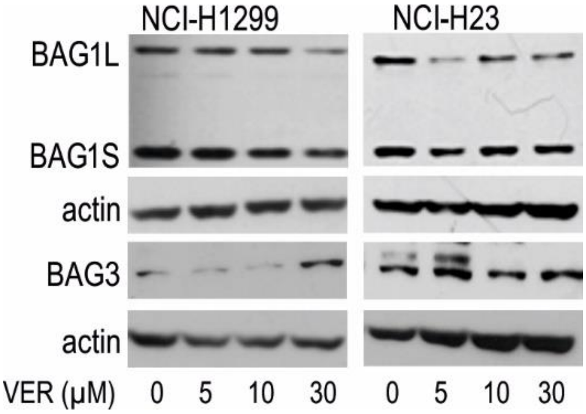

Figure S3. Western blot analysis of BAG-1 and BAG-3. Protein expression was analyzed in cells non-treated and treated with VER (24 h). Representative immunoblots are shown (n = 3), actin was used as a protein loading control.

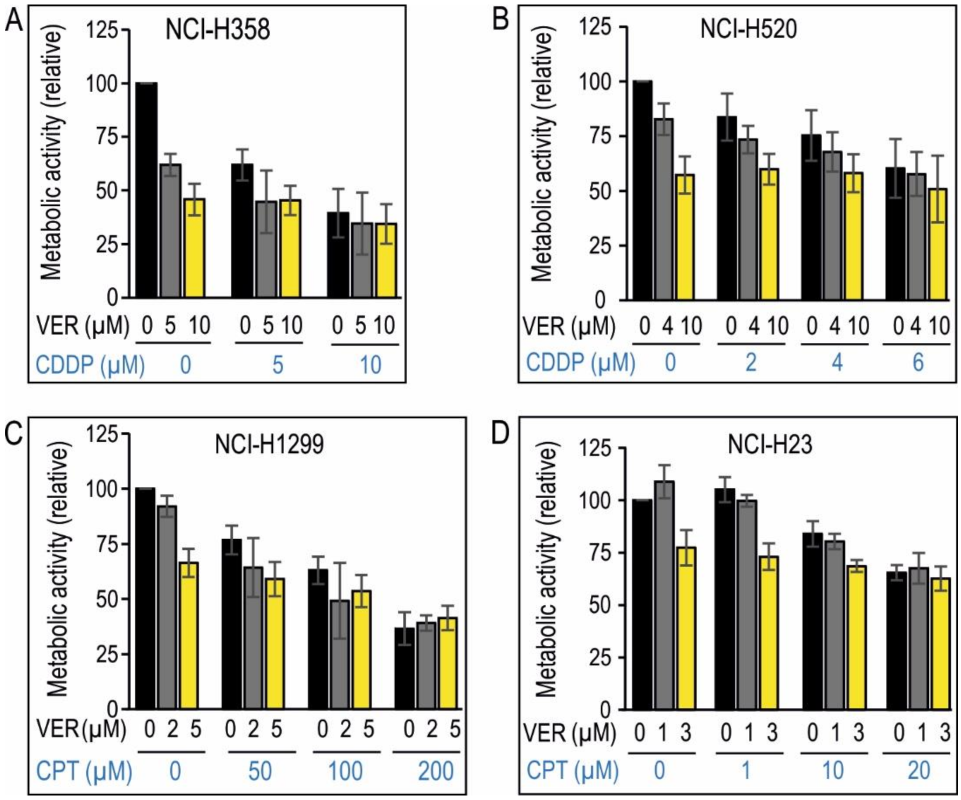

Figure S4. Viability of NSCLC cells following single or combined treatment with VER-155008 (VER) and platinum derivatives. (A-B) NCI-H358 (A) and NCI-H520 (B) were exposed to VER or cisplatin (CDDP) or a drug combination for 72 h. **(C-D)** NCI-H1299 (C) and NCI-H23 (D) cells were treated with VER or carboplatin (CPT) or a drug combination for 72 h. Cell viability was measured using MTS assay. Results are expressed in relation to the untreated control (mean ± SD, n = at least 3, each in triplicate).

Fig. 1B

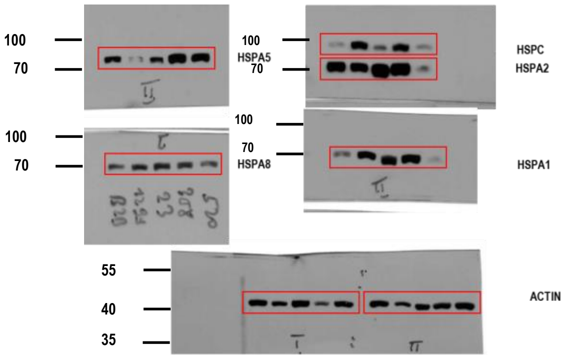

Fig. 1D

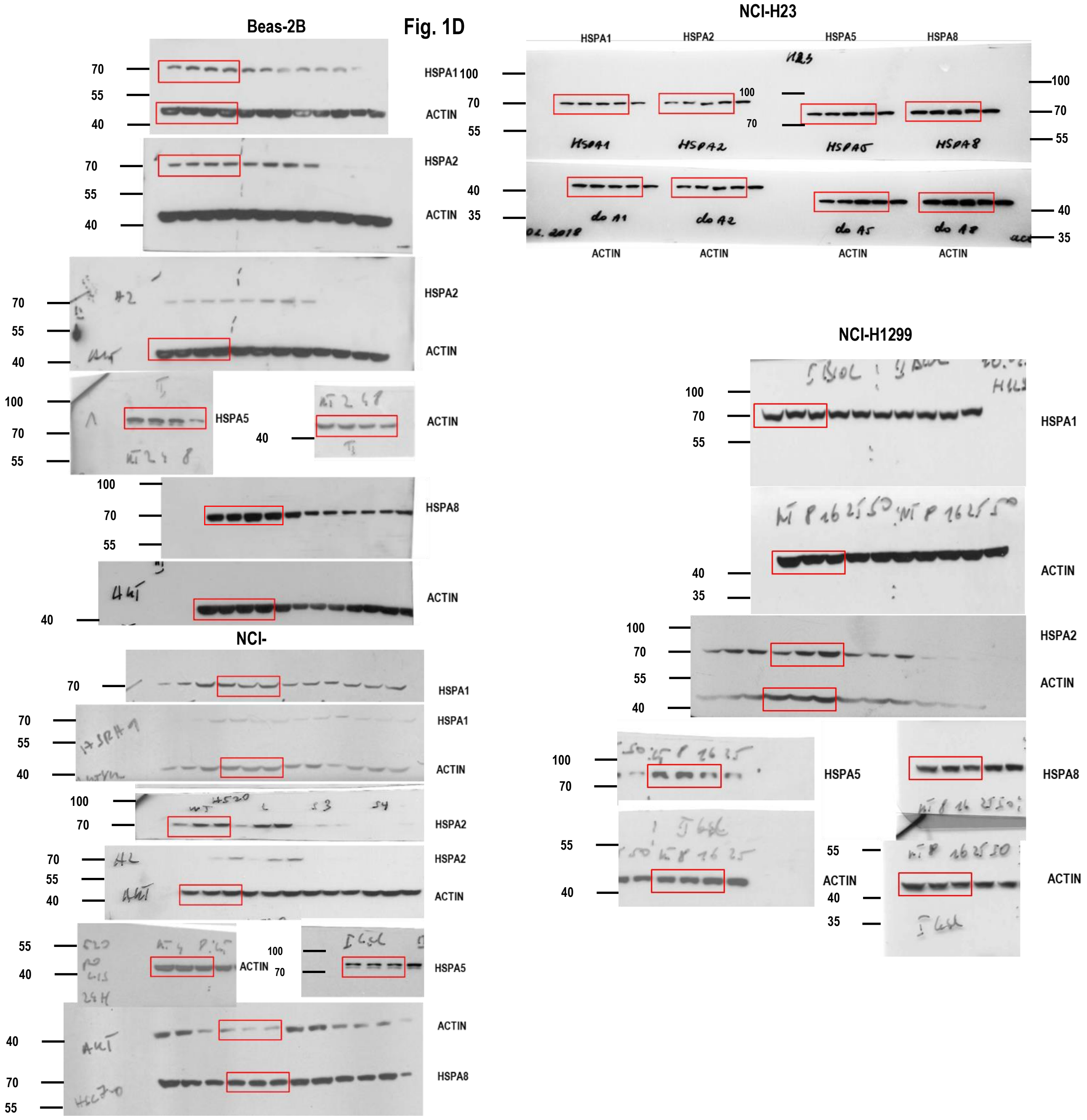

Figure S5. Unprocessed original scans of autoradiograms (immunoblots) included in Fig. 1B and 1D. Red lines identify cropped areas. In Fig. 1B the same membranes were used after incubation in Restore Western Blot Stripping Buffer (Thermo Fisher Scientific, Waltham, MA, USA).

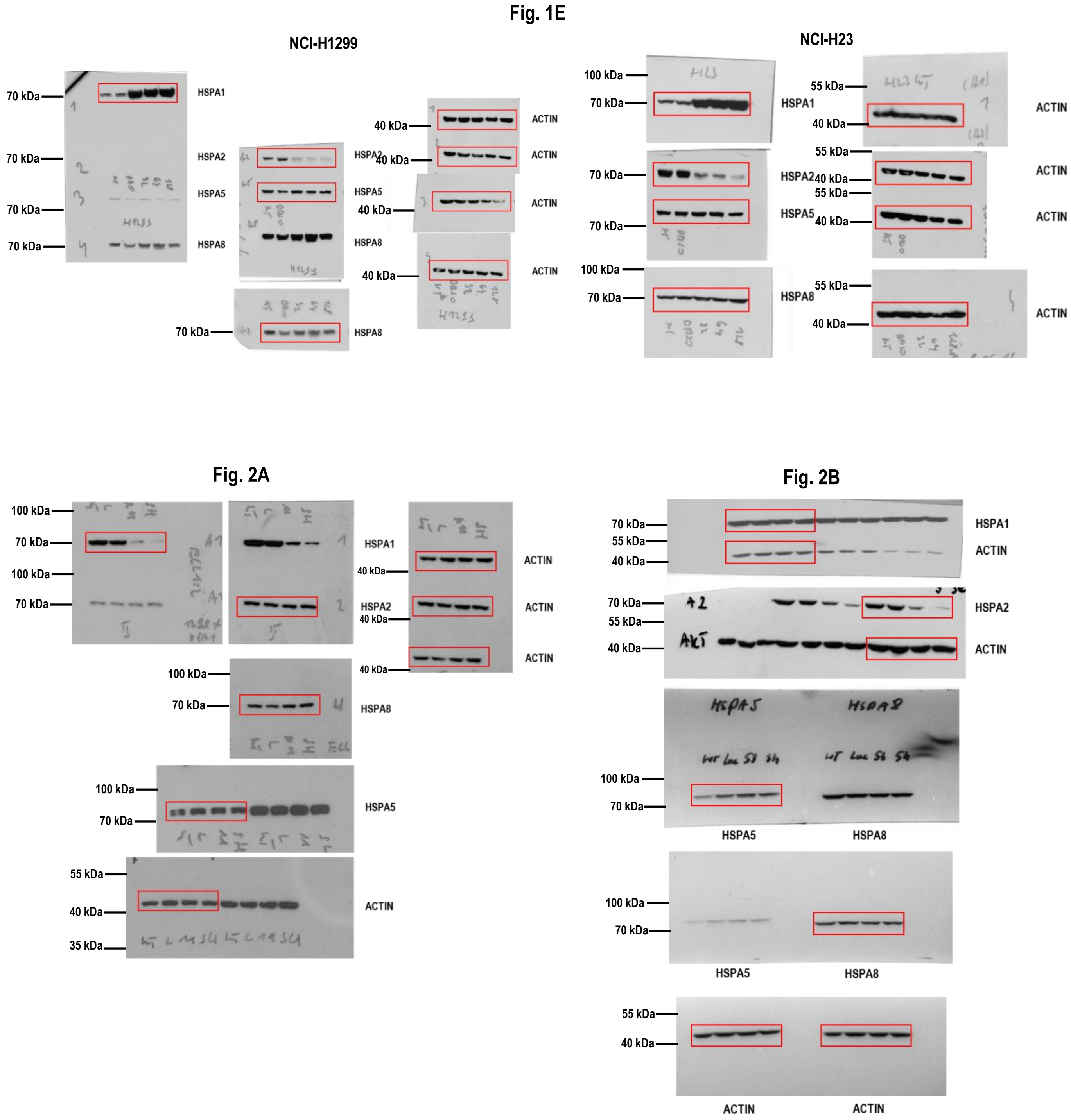

Figure S6. Unprocessed original scans of autoradiograms (immunoblots) included in Fig. 1E and Fig. 2. Red lines identify cropped areas.

Fig. 3A

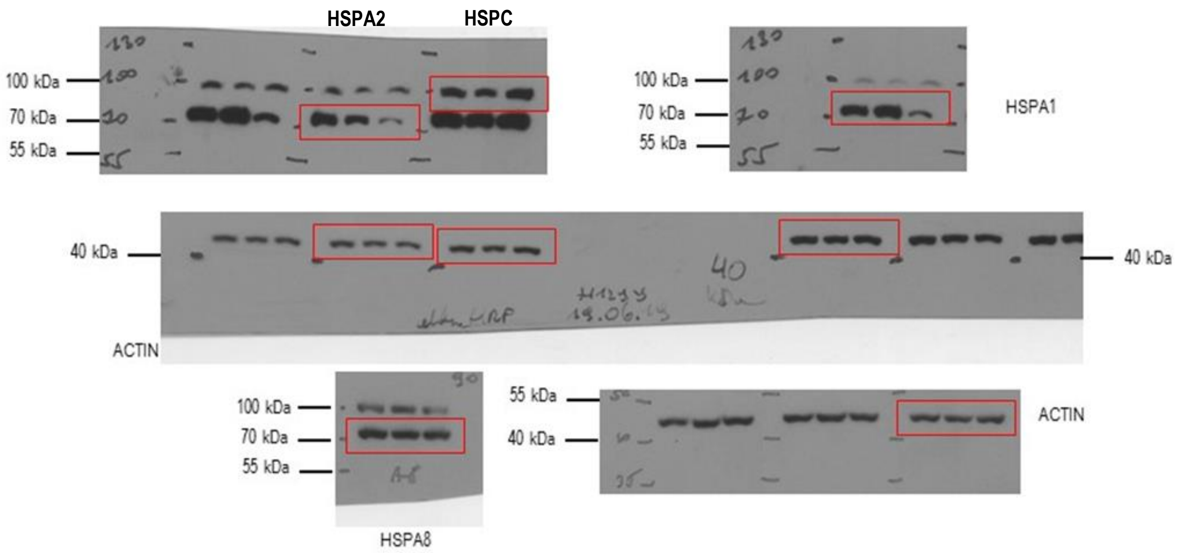

Figure S7. Unprocessed original scans of autoradiograms (immunoblots) included in Fig. 3A. Red lines identify cropped areas.

Fig. 4B

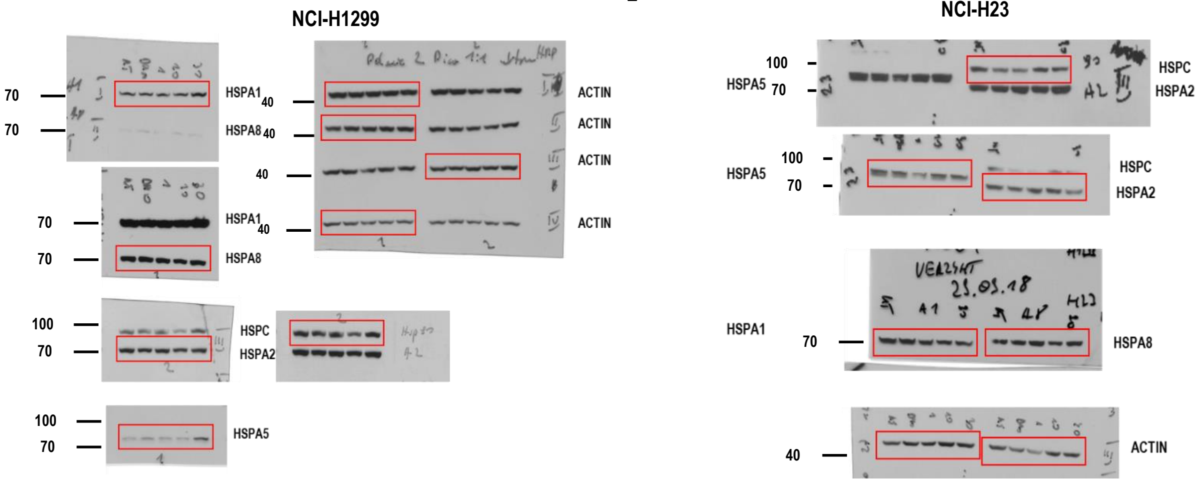

Fig. 4D

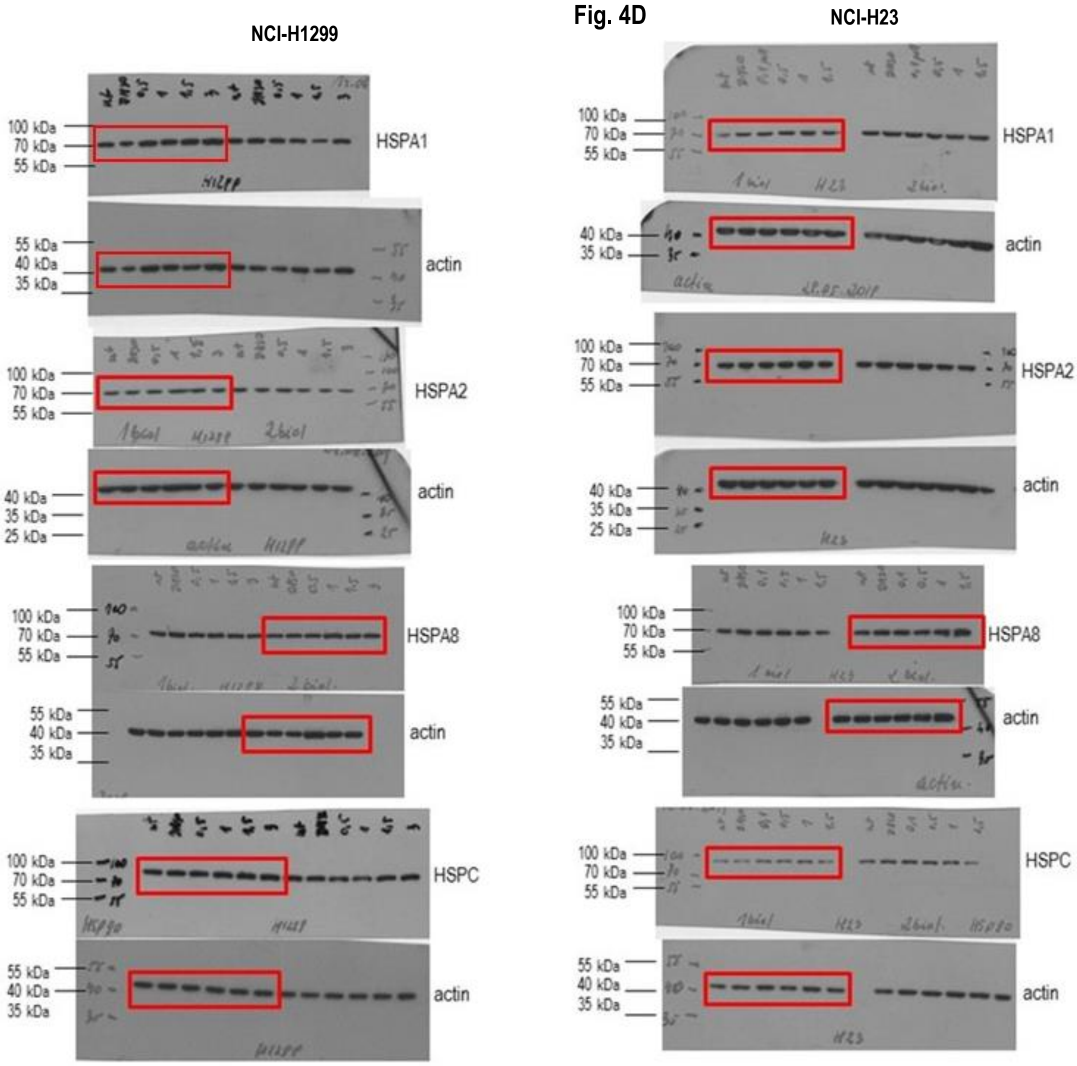

Figure S8. Unprocessed original scans of autoradiograms (immunoblots) included in Fig. 4B and Fig. 4D. Red lines identify cropped areas. In Fig. 4B the same membranes were used after incubation in Restore Western Blot Stripping Buffer (Thermo Fisher Scientific, Waltham, MA, USA).

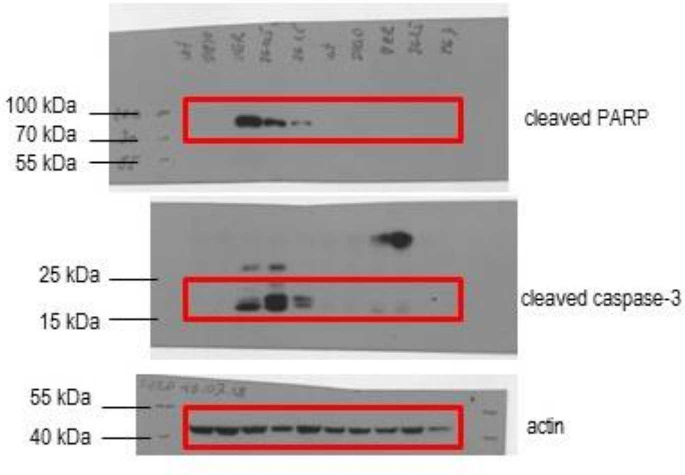

Figure S9. Unprocessed original scans of autoradiograms (immunoblots) included in Fig. 5G.

Red lines identify cropped areas.

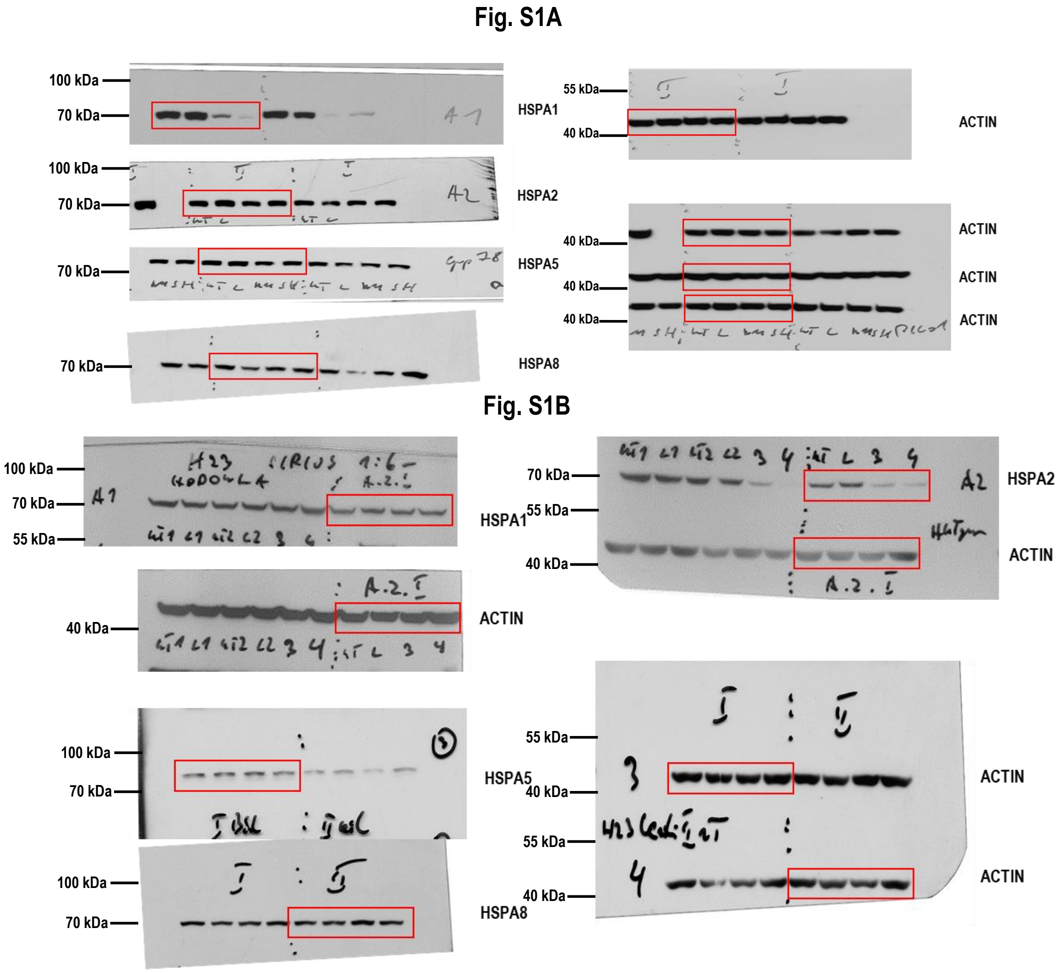

Figure S10. Unprocessed original scans of autoradiograms (immunoblots) included in Fig. S1.

Red lines identify cropped areas.

Fig. S3

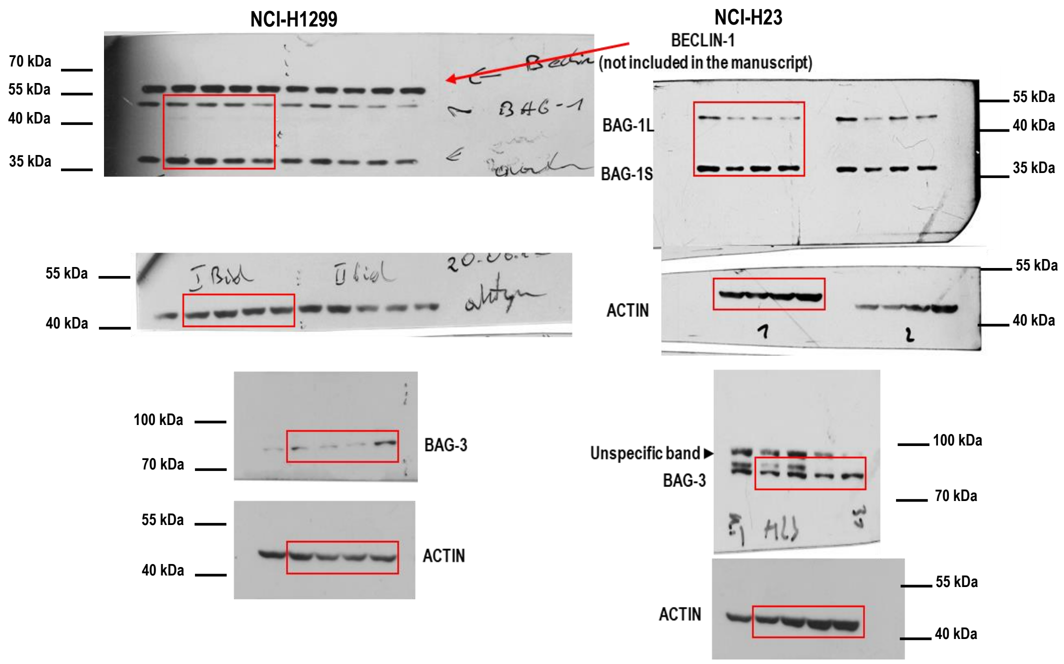

Figure S11. Unprocessed original scans of autoradiograms (immunoblots) included in Figure S3. Red lines identify cropped areas.
